# DNA Methylation and Transcriptomic Profiles of Wilms Tumour Reveal New Deregulated Genes and Epigenetic Processes Relevant for Tumour Stratification and Management

**DOI:** 10.1002/ijc.70680

**Published:** 2026-07-30

**Authors:** Abu Saadat, Francesco Cecere, Federica Rossetti, Laura Pignata, Emilia D'Angelo, Carlo Giaccari, Gaetano Verde, Paola Quarello, Franca Fagioli, Josep Roma, Manel Esteller, Enrique de Álava, Flavia Cerrato, Andrea Riccio, Angela Sparago

**Affiliations:** ^1^ Department of Environmental Biological and Pharmaceutical Sciences and Technologies (DiSTABiF) University of Campania “Luigi Vanvitelli” Caserta Italy; ^2^ Institute of Genetics and Biophysics (IGB) “Adriano Buzzati‐Traverso”, Consiglio Nazionale delle Ricerche (CNR) Naples Italy; ^3^ UOS Laboratori di Ricerca e Biobanca, UOC Ricerca Clinica e Traslazionale, AORN Santobono‐Pausilipon Naples Italy; ^4^ Department of Genetics, Genomics and Informatics University of Tennessee Health Science Center Memphis Tennessee USA; ^5^ Pediatric Onco‐Haematology Division Regina Margherita Children's Hospital Turin Italy; ^6^ Department of Public Health and Pediatrics University of Turin Turin Italy; ^7^ Childhood Cancer and Blood Disorders, Vall d'Hebron Research Institute (VHIR) Barcelona Spain; ^8^ Cancer Epigenetics Group Josep Carreras Leukaemia Research Institute (IJC) Badalona, Barcelona Catalonia Spain; ^9^ Centro de Investigacion Biomedica en Red Cancer (CIBERONC) Madrid Spain; ^10^ Institucio Catalana de Recerca i Estudis Avançats (ICREA) Barcelona Catalonia Spain; ^11^ Physiological Sciences Department, School of Medicine and Health Sciences University of Barcelona (UB) Barcelona Catalonia Spain; ^12^ Institute of Biomedicine of Sevilla (IBiS), Virgen del Rocio University Hospital/CSIC/University of Sevilla/CIBERONC Seville Spain; ^13^ Department of Normal and Pathological Cytology and Histology, School of Medicine University of Seville Seville Spain

**Keywords:** DNA methylation profile, epigenetic signature, *IGF2*, transcriptomic profile, Wilms tumour

## Abstract

Wilms Tumour (WT), the most common kidney cancer in children, presents features of altered kidney development and frequently exhibits molecular alterations at the 11p15.5 imprinted locus, affecting the *IGF2* and *H19* genes, which contribute to tumour growth and predisposition. The epigenetic landscape beyond 11p15.5 suggests diagnostic and prognostic potential, but its link to transcriptomic changes is largely unexplored. We integrated methylomic and transcriptomic datasets of 27 primary tumours and matched non‐neoplastic kidneys. DNA methylation profiling identified around 9000 differentially methylated CpG sites distinguishing neoplastic from non‐neoplastic tissue and other paediatric cancers, thus representing a novel WT‐specific epigenetic signature. We found that cases with Imprinting Centre 1 (IC1) gain of methylation exhibited the most extensive epigenetic alterations; cases with 11p15.5 loss of heterozygosity showed intermediate changes, whereas regressive tumours with largely normal 11p15.5 status were less affected. Three methylation clusters corresponding to transcriptomic subtypes were identified, characterised by distinct tumour microenvironment and chemosensitivity predictions: a regressive‐enriched, immune‐infiltrated group predicted to respond to paclitaxel, a proliferative group sensitive to doxorubicin, and a stromal‐like intermediate group. Combined analysis of methylation and expression data revealed more than 900 genes under epigenetic control, which contributed to defining the WT subtypes. Analysis of the *IGF2*/*H19* locus uncovered multiple regulatory mechanisms underlying *IGF2* activation, including imprinting defects at IC1, methylation changes at DMR0, differential promoter usage and transcriptional modulation by PLAG1 and BAHD1. These findings define the epigenetic alterations underlying WT heterogeneity and support improved molecular stratification and therapeutic approaches.

## Abstract (highlights)

What's new?

Wilms tumour is a paediatric kidney cancer commonly associated with 11p15.5 imprinting defects affecting *IGF2* and *H19*. By integrating methylomic and transcriptomic data from 27 tumours and non‐neoplastic kidneys, the authors identified ~9000 differentially methylated CpG sites specific to Wilms tumour and three methylation clusters that align with transcriptomic subtypes, each displaying distinct microenvironmental features and predicted chemosensitivity. Over 900 genes were found to be epigenetically regulated, and *IGF2* activation was controlled by multiple regulatory layers. These findings refine the molecular characterisation of Wilms tumour and provide a framework for future studies on prognostic stratification and therapeutic targeting.

## Graphical abstract

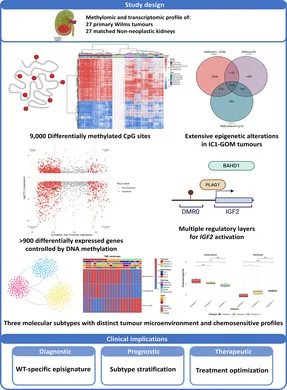

**Abbreviations**
BWSp: Beckwith‐Wiedemann Spectrum
DEG: Differentially Expressed Genes
DMRs: Differentially Methylated Regions
DMS: Differentially Methylated CpG Sites
GOM: Gain Of Methylation
IC1: Imprinting Centre 1
IC2: Imprinting Centre 2
LOH: Loss Of Heterozygosity
LOM: Loss Of Methylation
MatDel: Maternal 11p15.5 Deletion
ncDEG: negatively correlated Differentially Expressed Genes
NNK: Non‐Neoplastic Kidney
Norm11p15.5: Normal methylation and genotype at 11p15.5
PCA: Principal Component Analysis
PERMANOVA: Permutational multivariate Analysis Of Variance
SIOP: International Society of Paediatric Oncology
WT: Wilms Tumour

## 1 Introduction

Wilms Tumour (WT), or nephroblastoma, is the most common renal tumour in infants and children, characterised by abnormal kidney development [1]. WT diagnosis relies on histological examination, which usually results in an undifferentiated blastemal component with a variable amount of epithelial and stromal elements [2]. When only one cellular component is present, differential diagnosis with other kidney neoplasms or embryonal tumours is needed. WT progresses through the following stages: localised disease (Stages I–II), residual disease confined to the abdomen, including lymph node involvement, tumour spillage (pre‐ or intra‐operative), tumour rupture, or incomplete surgical resection (Stage III) and metastatic disease involving distant organs (Stage IV) [3]. These stages guide treatment and prognosis. In Europe, WT patients are treated under the International Society of Paediatric Oncology (SIOP) protocol, which involves preoperative chemotherapy, surgery and adjusted chemotherapy or addition of radiotherapy in high‐risk cases (blastemal, diffuse anaplastic tumours) [4]. The post‐treatment histological appearance may reveal changes, such as necrosis, fibrosis, macrophage infiltration, or inflammatory features, accounting for more than 66% of the total tumour volume in regressive cases and up to 100% in completely necrotic tumours [5]. Thanks to these therapies, patient survival rates have risen to over 90% for localised disease and 75% for metastatic disease, depending on histological subtype and tumour stage [6]. However, about 20% of patients relapse after first‐line therapy, and up to 25% of survivors report severe late morbidity from treatments [7, 8, 9].

The tumour usually arises sporadically in one kidney [10]. However, 5%–10% of patients have bilateral (Stage V) or multifocal tumours with earlier onset, 1%–2% have a family history, and up to 17% occur in genetic predisposition syndromes [6]. These include Beckwith‐Wiedemann Spectrum (BWSp), WAGR syndrome, Denys–Drash syndrome and Perlman syndrome, all associated with developmental defects and increased WT susceptibility. Although next‐generation sequencing studies have increased the number of genes associated with WT, their variants affect only a small subset of patients [11]. In contrast, DNA methylation changes are early and common events in Wilms tumourigenesis [12].

Most reported molecular lesions (~70% of cases) involve abnormalities at the chromosome 11p15.5 region [6]. These anomalies occur through either Gain Of Methylation (GOM) of the maternal copy of the Imprinting Centre 1 (IC1) or loss of the maternal allele with a copy number‐neutral effect (Loss Of Heterozygosity (LOH) or uniparental disomy) [13]. In the latter case, loss of methylation of the maternal copy of the Imprinting Centre 2 (IC2) is also observed. Notably, constitutional epigenetic defects at 11p15.5 cause BWSp [14, 15]. These molecular changes affect the expression of imprinted genes regulated by IC1, including maternally expressed *H19* and paternally expressed *IGF2*. *H19* produces a long non‐coding RNA that may influence cell proliferation, with its role in cancer being complex and context‐dependent [16]. *IGF2* encodes an embryonic growth factor controlling development and proliferation and is upregulated in many childhood and adult cancers [17]. Its expression is controlled by various promoters (P0 to P4) and multiple Differentially Methylated Regions (DMRs), including IC1 [18].

The availability of high‐throughput screening identified epigenetic defects beyond 11p15.5, proposed as biomarkers for WT. Charlton et al. found three WT‐specific methylation regions tested as circulating tumour DNA biomarkers in a pilot study [19]. Furthermore, epigenetic dysregulation profiles have been used for WT subclassification in a North American study [20]. However, the functional implications of these epigenetic changes remain unclear, and it is not well understood how they interact with methylation defects at 11p15.5 in WT development. Little is known about the clinical impact of the association between transcriptomic and methylomic profiles, and only one study has identified genes controlled by DNA methylation in WT [21]. Also, the mechanisms of *IGF2* activation in WT have been poorly investigated.

We used an extended analytical platform to define WT epigenetic architecture, study global gene‐expression changes and characterise alterations at the *IGF2/H19* locus. The results revealed a WT‐specific epigenetic signature, three molecular subtypes with distinct histology and tumour microenvironment, and shed light on the complex, multi‐layered regulation of *IGF2*.

## 2 Materials and Methods

### 2.1 Sample Acquisition

Twenty‐seven patients were recruited from the Pediatric Oncology Unit of the Institute of Biomedicine of Seville. All patients were treated with neoadjuvant chemotherapy (vincristine and actinomycin‐D) before nephrectomy according to the SIOP‐93‐01 and SIOP 2001 protocols. All non‐neoplastic kidneys (NNKs) were collected from renal tissue distant from the tumour in nephrectomy specimens, and tumour infiltration was previously excluded by H&E staining in a subset of samples [22]. Specimens, clinical and molecular features, together with omic analyses performed in this study, are reported in Table 1. Stages I and II tumours were merged into one group due to similar disease spread. The validation cohort consisted of 34 additional WT samples without matched NNK tissues, obtained from the Regina Margherita Children's Hospital in Turin.

**TABLE 1:** Wilms tumour cases in this study: Clinicopathological features, molecular alteration of the 11p15.5 region and performed omic analyses.

Sample	Clinical features				11p15.5 methylation		11p15.5 genotyping^a^	Molecular group^b^	OMIC analyses	
	Occurrence	Hystological type	Stage	Risk grade	IC1	IC2			EPIC array	RNA‐seq
WT6	UL	B (Anaplastic)	III	High	GOM	LOM	CN‐LOH	LOH	Y	Y
WT7	UL	R	II	Int	GOM	LOM	NA	LOH	Y	Y
WT8	UL	M	I	Int	GOM	LOM	CN‐LOH	LOH	Y	Y
WT9	UL	M	III	Int	GOM	LOM	CNV‐Del	MatDel	Y	Y
WT10	UL	M	I	Int	GOM	LOM	CN‐LOH	LOH	Y	NA
WT11	UL	E	I	Int	GOM	LOM	CN‐LOH	LOH	Y	Y
WT16	BL	Right: R; Left: B	V	High	GOM	LOM	CN‐LOH	LOH	Y	Y^d^
WT17	UL	M	III	Int	GOM	LOM	CN‐LOH	LOH	Y	NA
WT18	UL	S	I	Int	GOM	LOM	CN‐LOH	LOH	Y	Y
WT19	UL	R	I	Int	GOM	LOM	CNV‐Del	MatDel	Y	Y
WT23	UL	S	I	Int	GOM	n	n	IC1‐GOM	Y	Y^e^
WT24	UL	E	I	Int	GOM	n	n	IC1‐GOM	Y	Y
WT25^c^	UL	E	III	Int	GOM	n	n	IC1‐GOM	Y	Y
WT26^c^	BL	Right: B; Left: B	V	High	GOM	n	n	IC1‐GOM	Y	Y
WT27	UL	E	III	Int	GOM	n	n	IC1‐GOM	Y	Y
WT28	UL	R	I	Int	GOM	n	NA	IC1‐GOM	Y	Y
WT29	BL	Right: B; Left: R	V	High	GOM	n	n	IC1‐GOM	Y	Y
WT30	UL	S (Anaplastic)	II	Int	GOM	n	n	IC1‐GOM	Y	Y
WT31^c^	BL	Right: R; Left: E	V	Int	GOM	n	n	IC1‐GOM	Y	Y
WT32^c^	UL	B	III	High	GOM	n	n	IC1‐GOM	Y	Y
WT33	BF	S	III	Int	GOM	LOM	NA	LOH	Y	Y^d^
WT39	UL	R	II	Int	n	n	n	Norm11p15.5	Y	Y
WT40	UL	S	II	Int	n	n	n	Norm11p15.5	Y	Y
WT41	UL	R	I	Int	n	n	n	Norm11p15.5	Y	Y^e^
WT42	UL	R	I	Int	n	n	n	Norm11p15.5	Y	Y^d^
WT43	UL	M	III	Int	n	n	n	Norm11p15.5	Y	Y
WT48	UL	R	II	Int	n	n	n	Norm11p15.5	Y	Y

*Note:* NNK samples correspond to matched non‐neoplastic kidney tissues collected from the same nephrectomy specimens and used as paired controls. Y, analysed as matched WT‐NNK pair.

Abbreviations: B, blastimal; BF, bifocal; BL, bilateral; CN, copy neutral; CNV, copy number variant; E, epithelial; GOM, gain of methylation; IC1, Imprinting Centre 1; IC2, Imprinting Centre 2; Int, intermediate; LOH, loss of heterozygosity; LOM, loss of methylation; M, mixed; MatDel, maternal deletion; n, normal; NA, not assayed; NNK, non‐neoplastic kidney; R, regressive; S, stromal; UL, unilateral; WT, Wilms Tumour.

^a^ SNP‐array performed in ref. [23].

^b^ Based on molecular alterations in the 11p15.5 region.

^c^ Cases with molecular predisposition (IC1‐GOM detected in both WT and corresponding NNK tissues). The underlined text represents the histological type for the analysed side of bilateral WT cases.

^d^ WT‐only RNA‐seq sample.

^e^ NNK‐only RNA‐seq sample.

### 2.2 Nucleic Acids Extraction

DNA and RNA were extracted from fast‐frozen samples of WTs and NNKs using Phenol‐chloroform‐isoamyl and Trizol protocols, respectively.

### 2.3 DNA Methylation Profiling and Data Analysis

Genome‐wide methylation profiling was performed using Illumina's Infinium MethylationEPIC BeadChip array. The obtained IDAT files were imported into R with the champ.load function from the ChAMP (v2.34.0) package [24] with method set as ‘minfi’ and other parameters set to default. The beta values were normalised using BMIQ normalisation. CpG sites on the X and Y chromosomes were excluded to avoid potential sex‐related confounding effects. IC1 and IC2 methylation abnormalities were defined as methylation values outside the mean ±2.5 SD range calculated from NNK controls analysed in the same cohort (Table S1).

### 2.4 Total RNA‐Seq and Data Analysis

Transcriptome profiling was performed using total RNA sequencing. Sequencing libraries were prepared using Ovation SoLo RNA‐seq Library Preparation kit (Tecan Genomics, Redwood city, CA) as per the manufacturares instruction manual (library type: fr‐secondstrand). Reads were sequenced on paired‐end 150 bp mode on NovaSeq 6000 (Illumina, San Diego, CA). The obtained reads were assessed for quality using FastQC and trimmed for adapter sequences using Trimmomatic (v0.39) in paired‐end mode and ‘MINLEN’ parameter set to 50. Only the good quality reads were considered for alignment to the human reference genome (hg19) using STAR (v2.7.4a) [25] with ‘outFilterMultimapNmax’ to 1. UMI‐Tools [26] utility was employed in PE mode to remove the duplicate reads. The filtered reads were then assigned to genes using ‘featureCount’ function from Subread package (v2.0.1) [27]. Sequencing coverage and quality are detailed in Table S2.

### 2.5 Bioinformatic and Statistical Analyses

Detailed methods for bioinformatic and statistical analyses, including paired comparisons between tumour and control tissues using matched WT‐NNK samples from the same patient, are provided in Supporting Information: Supplementary Methods, together with a detailed description of the processing of publicly available datasets.

## 3 Results

### 3.1 Patient Cohort

The baseline characteristics of the 27 WT cases are summarised in Table 1. Stage and risk were assessed post‐surgery and pathological examination, according to SIOP classification [28]. Most patients had unilateral presentation. Four were bilateral, one bifocal. Tumours were mostly profiled in pairs with the corresponding NNK. Specifically, DNA methylation analyses were performed on all 27 matched WT‐NNK pairs, whereas transcriptomic analyses included 20 matched WT‐NNK pairs, 2 unpaired WT samples and 3 unpaired NNK samples (Table 1). Molecular subgroup stratification was established by IC1/IC2 methylation profiles (Table S1) and supported by available SNP arrays data [23]. Tumours showing isolated IC1 hypermethylation were classified as IC1‐GOM, whereas cases with combined IC1 hypermethylation and IC2 hypomethylation were classified as LOH when copy‐neutral and as maternal deletion (MatDel) when associated with a deletion. Tumours lacking detectable methylation or structural abnormalities were classified as Norm11p15.5. Notably, the NNK tissue of 4 patients was hypermethylated in IC1 compared to other NNK samples (*β*‐value > 0.7 vs. mean NKK = 0.579, Table S1) and were excluded from the calculation of mean IC1 methylation of controls. These cases, suggestive of molecular predisposition associated with somatic mosaicism for chromosome 11p15.5 alterations, may be compatible with either BWSp or isolated WT [29] and are indicated in Table **1**. In cancer tissues, 10/27 cases had IC1‐GOM, 9 had copy‐neutral (unipaternal) 11p15.5 LOH, 2 had maternal 11p15.5 deletion (MatDel) and 6 showed normal methylation and genotype at 11p15.5 (Norm11p15.5).

### 3.2 Comprehensive DNA Methylation Profiling Revealed Clustering of WT Histological and Molecular Types

To assess epigenetic changes in WT beyond 11p15.5 defects, we performed whole‐genome DNA methylation analysis on tumour and control samples using methylation array. Unsupervised principal component analysis (PCA) of all probes showed clear separation between controls and tumours, consistent with minimal tumour contamination in the majority of NNK samples. While NNK samples appeared relatively homogeneous, WT samples showed heterogeneity without clear segregation of stages or histological types (Figure 1A). Two tumour samples (WT25 and WT27) were grouped with the controls in agreement with the findings of Brzezinski et al. [20], who found a small group of tumours similar to mature kidney tissue in the PCA space and showing fewer molecular changes. Consistent with previous reports [30, 31], we found significantly lower global DNA methylation levels in tumours than in controls, indicating DNA methylation loss during tumuorigenesis (Figure S1A). After filtering for a mean difference of 30% and adjusted *p* value < 0.001, we found 8923 Differentially Methylated CpG Sites (DMSs), of which 4179 were hypermethylated and 4744 hypomethylated in tumours compared to controls (Figure 1B, Table S3). 36.5% of the DMSs (3257/8923) were located in intergenic regions and 61.6% of them were hypomethylated (Figure 1C). In addition, 36.2% (3234) of the DMSs corresponded to gene bodies and 27% (2412) to promoters, these ones mostly hypermethylated (Figure 1C, Table S3).

**FIGURE 1:**
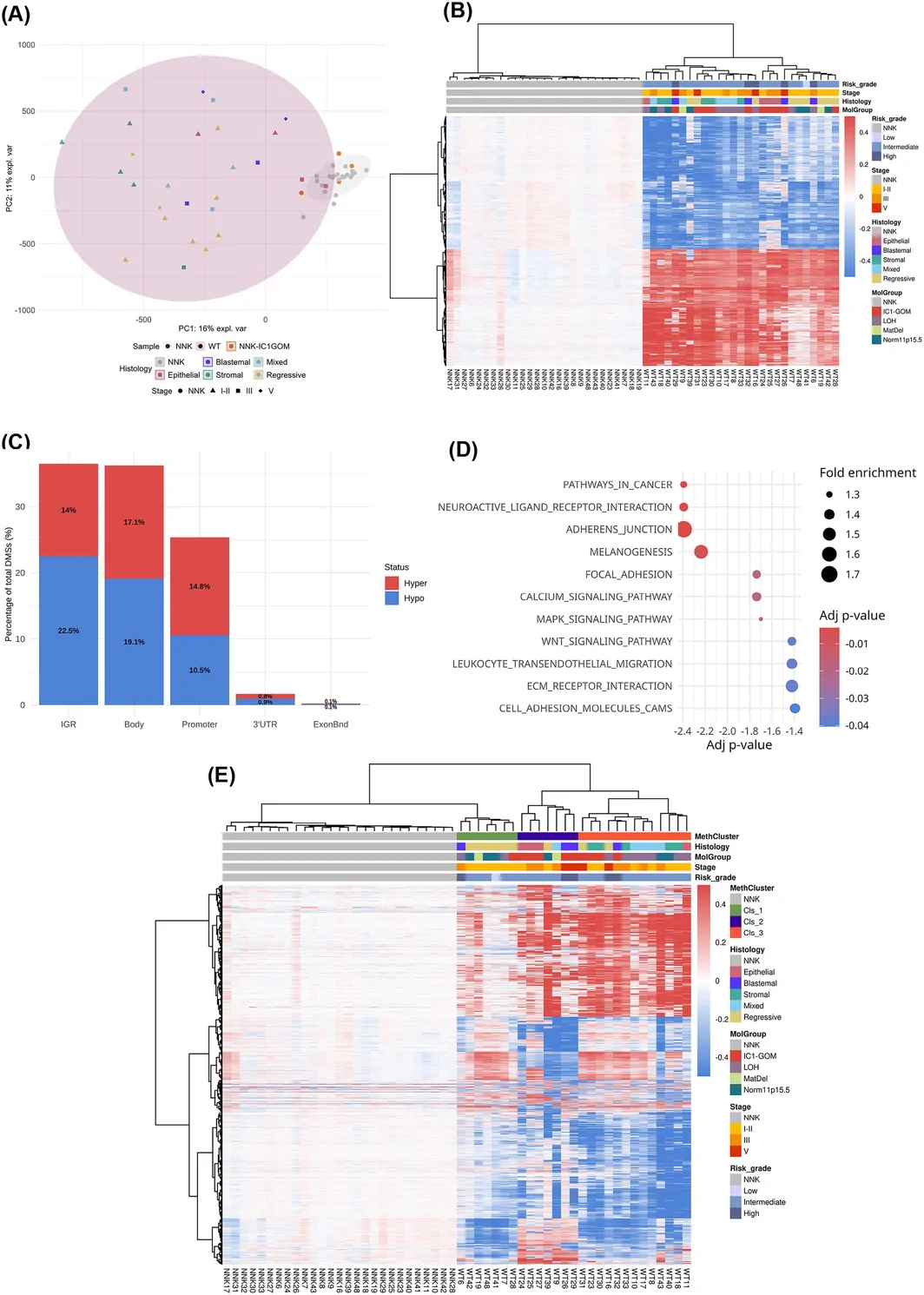
Profile of DNA methylation alterations in WT. (A) PCA of the genome‐wide DNA methylation profiles, performed using beta values from all 734,767 CpG sites (post filtering) profiled by the Illumina EPIC array. Each point represents an individual sample. The first principal component (PC1, x‐axis) explains 16% of the total variance, while the second principal component (PC2, y‐axis) accounts for 11% of the variability. Samples are colour‐coded by histological subtypes and shaped according to tumour stage. (B) Heatmap showing the hierarchical clustering of 8923 DMSs. Each column represents a sample, and each row a CpG site. The colour scale represents the Delta Beta‐values, calculated by normalising to the control mean. (C) Genomic distribution of DMSs across major genomic features. The promoter was characterised by probes overlapping the 5′UTR, TSS1500, TSS200 and 1st Exon regions. (D) KEGG pathway enrichment analysis of DMSs in the WT episignature. Each dot represents a significantly enriched pathway. The fold enrichment (dot size) indicates the number of affected genes in that pathway; the significance (from blue to red) is based on the adjusted *p* value from hypergeometric testing. (E) Heatmap of the top 5000 most variable CpG sites among WT samples. Variance was calculated by comparing each sample's beta value to the mean methylation level for the corresponding CpG site. Each column represents 1 sample and each row represents 1 of the top 5000 most variable probes. Three subsets were identified by clustering and are indicated above the heatmap. The colour scale represents the Delta Beta‐values, calculated by normalising to the control mean.

To assess the functional significance of the methylation profile, we performed a KEGG pathway analysis of all genes annotated to DMSs (Figure 1D, Table S4). The most significant term was ‘pathways in cancer’, indicating an important role of methylation changes in WT development. We identified DNA methylation alterations in the promoters of cancer pathway genes (https://www.kegg.jp/pathway/hsa05200), involving both tumour suppressors (e.g., *CTNNA1* and *PPARG*) and oncogenes (e.g., *FGFR1* and *IL7R*), as shown in Figure S1B. Significant enrichment was also revealed for pathways related to cell adhesion and tumour microenvironment interactions, including ‘adherens junction’, ‘focal adhesion’, ‘ECM‐receptor interaction’ and ‘cell adhesion molecules’. In addition, signalling pathways frequently implicated in tumour progression, such as Wnt, MAPK and calcium signalling, were enriched among genes affected by methylation changes. ‘Neuroactive ligand receptor interaction’, was also significantly enriched due to hypomethylation in many olfactory receptor genes. The latter phenomenon is likely due to their genomic localisation, mainly in heterochromatic subtelomeric and pericentromeric regions, which frequently undergo DNA methylation loss in cancer [32].

Unsupervised hierarchical clustering of all DMSs did not reveal a clear segregation of tumour samples by histotype (Figure 1B). However, partial proximity among some tumours with similar histology was observed, particularly within the WT7‐WT28 sample range. Consistently, PERMANOVA and dispersion tests revealed significant differences between regressive‐ and epithelial‐type tumours and other histotypes, either analysing all DMSs (adjusted *p* values 0.011988 and 0.028970, respectively; Table S5) and focusing on DMSs in the promoters of cancer‐related pathway genes (adjusted *p* values 0.007992 and 0.01598, respectively; Table S5). The non‐significant beta‐dispersion results indicated that these differences were not driven by unequal within‐group variability.

To understand WT stratification, we performed an unsupervised hierarchical clustering of the top 5000 variable CpGs (Figure 1E). We identified three main subsets: Cluster 1 (*n* = 7/27 [26%]), Cluster 2 (*n* = 7/27 [26%]) and Cluster 3 (*n* = 13/27 [48%]). Notably, Cluster 1 tumours clustered with controls and mainly consisted of regressive‐type samples with fewer epigenetic changes. Also, some epithelial‐type tumours in Cluster 2, including WT25 and WT27 samples, exhibited minimal alterations. PERMANOVA showed significant methylation differences in regressive (*p* = 0.03796) and epithelial (*p* = 0.04795) tumours versus other histological subtypes. Fisher's test confirmed the overlap between methylation clusters and histological types (*p* = 0.00695), demonstrating that methylation‐based clustering aligns with histology. A borderline association was observed between methylation clusters and 11p15.5 molecular groups (*p* = 0.07391). In particular, Norm11p15.5 tumours were more represented in Cluster 1, while IC1‐GOM and LOH cases were more represented in Clusters 2 and 3, respectively, although with partial overlap among clusters (Figure 1E). Notably, the four NNK samples displaying IC1 hypermethylation (NNK25, NNK26, NNK31 and NNK32) clustered together with the other NNK controls and did not group with tumour samples. Consistently, PCA of global methylation profiles confirmed their similarity to controls (Figure S1C), indicating the absence of widespread epigenetic disruption in NKK samples from cases with IC1‐GOM predisposition.

In summary, whole‐genome methylation profiling revealed widespread epigenetic alterations beyond 11p15.5 and highlighted heterogeneous methylation patterns partially associated with histological and molecular WT features.

### 3.3 Methylation Profiles of 11p15.5 Molecular Subgroups

Given the key role of the 11p15.5 locus in Wilms' tumourigenesis, we further analysed the whole‐genome methylation profiles of WTs with different 11p15.5 molecular changes. Since there were only two 11p15.5 MatDel samples, we excluded them from further analyses. We identified subgroup‐specific DMSs by comparing each tumour group to controls (mean difference 30% and adjusted *p* value < 0.001). Notably, IC1‐GOM tumours exhibited more DMSs than LOH and Norm11p15.5 cases (Figure 2A). They also exhibited the highest number of changes across all genomic elements, including both hypermethylation and hypomethylation in promoters, gene bodies and intergenic regions (Figure 2B). Although distinct epigenetic profiles among the 11p15.5 subgroups were not clearly separated, subgroup‐specific DMSs showed the strongest methylation differences in the subgroup in which they were identified, while maintaining directionally consistent but attenuated changes across the remaining subgroups, supporting a continuum rather than fully discrete subtype‐specific methylation patterns (Figure 2C). In particular, the IC1‐GOM subgroup showed more homogeneous methylation changes than other groups. Notably, regressive histology was significantly enriched in the Norm11p15.5 subgroup (Fisher's Exact Test *p* value 0.04413, Figure 2C). KEGG pathway analysis based on subgroup‐specific methylation changes identified cancer‐related pathways in all molecular subgroups, although enrichment was more extensive and statistically significant in IC1‐GOM tumours (Table S6, Figure 2D). Gene expression analysis of DNA methylation regulators across the three molecular subgroups revealed a general upregulation of these factors in tumour samples compared to controls, with *DNMT3B* as the only gene showing limited or no significant changes (Figure 2E). In particular, *DNMT3A* and *TET2* exhibited the highest expression levels in the IC1‐GOM subgroup, highlighting a coordinated dysregulation of the DNA methylation machinery in WT, most evident in IC1‐GOM tumours.

**FIGURE 2:**
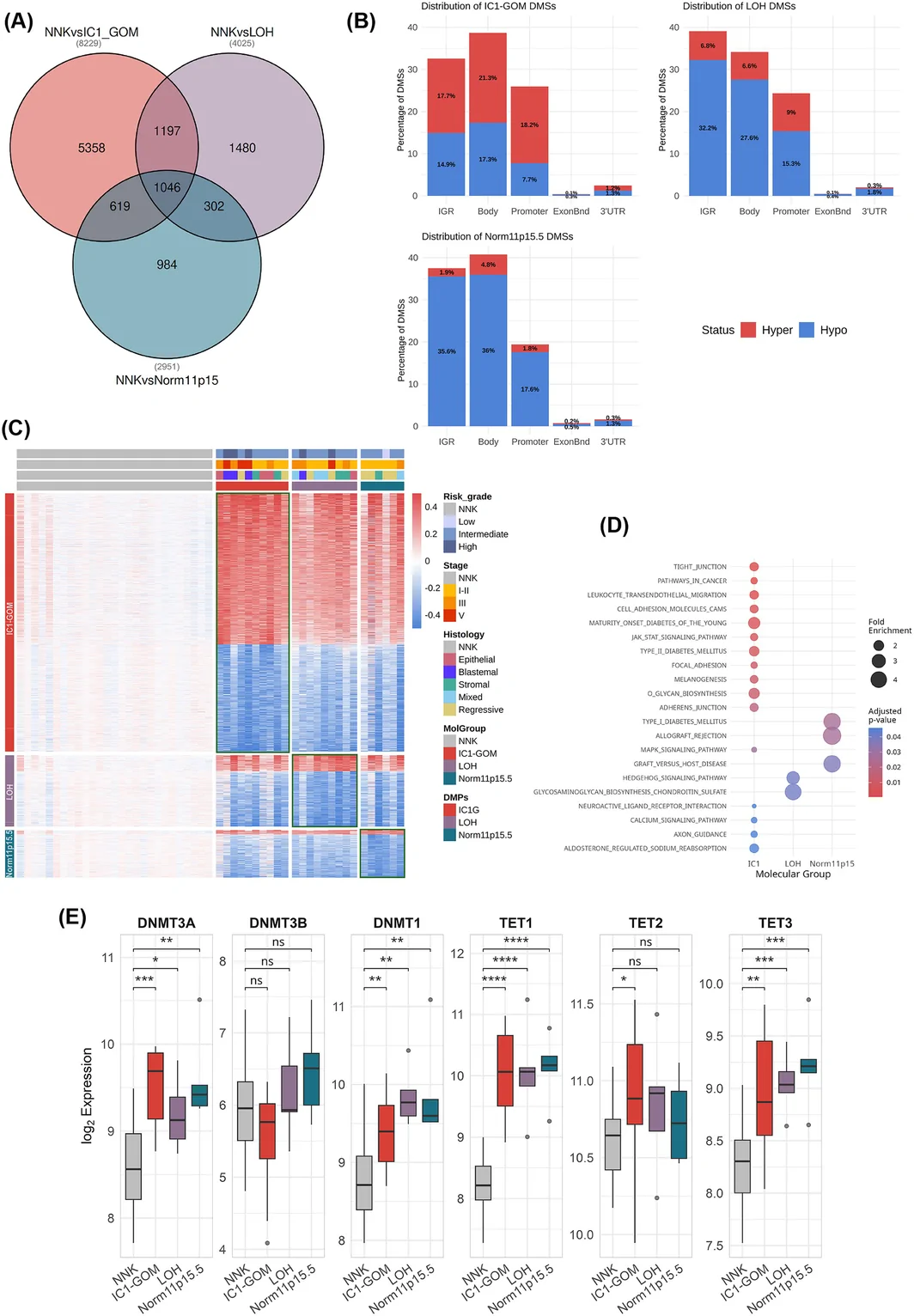
Impact of distinct 11p15.5 alterations on global DNA methylation. (A) Venn diagram illustrating the number of exclusive and shared DMSs among WT subgroups, stratified by molecular alterations in the 11p15.5 region. DMSs were identified within each group relative to NNKs using a mean methylation difference ≥ 30% and adjusted *p* value < 0.001. (B) Genomic distribution of subgroup‐specific DMSs across major genomic features in the three WT subgroups. The promoter was characterised by probes overlapping the 5′UTR, TSS1500, TSS200 and 1st Exon regions. (C) Comparison among subgroup‐specific DMSs. Columns represent tumour samples ordered according to the 11p15.5 molecular group, whereas rows represent subgroup‐specific DMSs (green squares), grouped according to the molecular subgroup in which they reached statistical significance and arranged within each group by decreasing magnitude of methylation change. (D) KEGG pathway enrichment analysis of DMSs in the three WT subgroups. (E) Expression of DNA methylation regulators across WT subgroups. Boxplots showing log_2_‐normalised expression levels of DNMT and TET family members in NNK controls and WT molecular subgroups. Statistical significance is indicated as follows: ns, not significant; *p < 0.05; **p < 0.01; ***p < 0.001; ****p < 0.0001.

To assess whether alterations are confined to the 11p15.5 locus or reflect broader imprinting defects capable of stratifying samples, we analysed DNA methylation and transcriptomic changes across known imprinted regions. Although widespread alterations in imprinting methylation and gene expression were observed in tumours compared to controls, no major differences emerged across 11p15.5 molecular subgroups. Recurrent DNA methylation changes affected multiple loci, including hypermethylation at *RB1*:int2‐DMR, *ZNF597*:3′‐DMR, *INPP5F*:Int2‐DMR and *SNRPN*:int1‐DMR1, and hypomethylation at *MAGEL2*:TSS‐DMR and *ERLIN2*:Int6‐DMR (Figure S2A), with some samples (e.g., WT11 and WT39) showing greater perturbation. As expected, IC1‐GOM tumours also exhibited alterations at secondary *IGF2* DMRs, whereas the LOH group showed IC2 hypomethylation. Notably, some additional DNA methylation changes at imprinted loci were observed in IC1‐GOM NNK samples, particularly NNK25 and NNK26, although these alterations appeared sporadic and non‐homogeneous (Figure S2A). At the transcriptional level, imprinted gene expression displayed considerable inter‐sample variability, including among controls, with no clear subgroup‐specific patterns (Figure S2B). Nevertheless, several loci (e.g., *MEG* genes, *PLAGL1*, *UBE3A*, *RB1*, *PEG3* and *PEG10*) were markedly dysregulated, particularly in IC1‐GOM tumours. Notably, IC1‐GOM NNK samples, and most prominently the corresponding tumours (WT25, WT26, WT31 and WT32), exhibited pronounced alterations at the *SNURF‐SNRPN* locus. Our findings indicate WTs with IC1‐GOM had the broadest, cancer‐associated methylation alterations.

### 3.4 Definition of a WT‐Specific Epigenetic Signature

To validate DNA methylation alterations, we analysed 34 further WT methylomes derived from an Italian cohort. After intersecting 696,485 CpGs across datasets, 8147 of the 8923 DMSs identified in the study cohort (91%) showed concordant differential methylation in the validation cohort. PCA performed using these shared DMSs confirmed the separation of WT samples from both cohorts relative to NNK controls from the study cohort (Figure 3A). Since only minor differences were found between the study and validation cohorts, and the latter included lower‐quality samples, we performed subsequent analyses with the original 8923 DMSs. To further assess the specificity of the WT episignature beyond matched NNK controls, we compared these DMSs with second‐trimester foetal kidney methylomes. PCA and hierarchical clustering showed that 4427 CpG sites shared between the EPIC and 450 K platforms effectively distinguished normal foetal tissues from tumours (Figure 3B).

**FIGURE 3:**
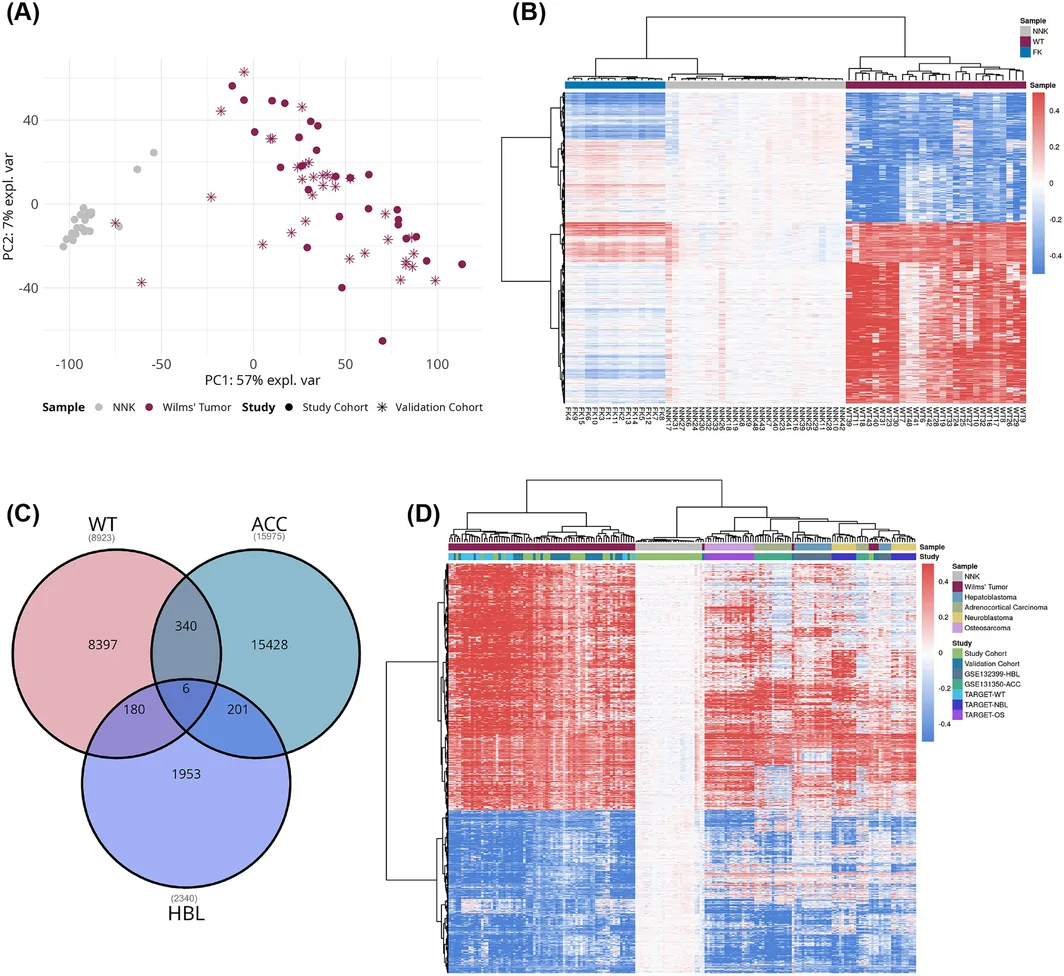
Validation of the WT‐specific episignature. (A) PCA of shared 8147 CpG sites, corresponding to DMSs in study cohort and confirmed in an independent Italian validation cohort of WTs profiled by the Illumina EPIC array. (B) Hierarchical clustering of WT and foetal kidney (GSE69502) methylation profiles. The plots refer to 4427 DMSs as a refinement between two distinct arrays. (C) Venn diagram showing the overlap among epigenetic signatures in WT, adrenocortical carcinoma and hepatoblastoma, respectively. For each tumour, the total number of DMSs is indicated in brackets. DMSs were identified within each group relative to controls using a mean methylation difference ≥ 30% and adjusted *p* value < 0.001. (D) Heatmap showing the comparison between WT and other paediatric tumours. A reduced set of 3908 DMSs was used for the analysis, including WT and NNK samples from the study cohort, WT samples from the validation cohort and the TARGET dataset, as well as hepatoblastoma (HBL), adrenocortical carcinoma (ACC), neuroblastoma (NBL) and osteosarcoma (OS) datasets.

To evaluate whether the identified DMSs represented a WT‐specific epigenetic signature rather than a general feature of paediatric embryonal tumours with imprinting alterations, we compared them with public methylation datasets of hepatoblastoma and adrenocortical carcinoma. We calculated the tumour‐specific DMSs of these neoplasms using the same strict criteria applied to our study cohort. The intersection of the obtained methylation profiles demonstrated the specificity of the vast majority of the WT DMSs (94%, 8397), indicating the distinctive nature of these CpGs as a novel and extended WT episignature (Figure 3C). We confirmed the specificity of these DMSs by unsupervised hierarchical clustering, including TARGET datasets for neuroblastoma, osteosarcoma, and further WT samples, generated using a smaller 450 K array. The results definitely showed that the shared WT‐associated DMSs not only differentiate WTs from NNKs but also segregate these neoplasms from other paediatric tumours profiled using independent methylation datasets (Figure 3D).

In summary, validation with other cohorts confirmed the DMSs as robust, tumour‐specific features, defining a distinctive WT epigenetic signature.

### 3.5 Transcriptomic Profiling Revealed WT‐Specific Deregulated Genes Involved in Cancer Processes and Kidney Functions

Transcriptomic analysis on NNK‐WT pairs identified 4673 Differentially Expressed Genes (DEGs) after filtering for log_2_ fold change ±1 and *p* value 0.05. Of these, 38.5% (1800) were upregulated and 61.5% (2873) were downregulated (Figure S3A, Table S7). In line with epigenetic changes at 11p15.5, tumours showed increased *IGF2* and decreased *H19* expression. Notably, *IGF2* activation was observed across all 11p15.5 molecular subgroups compared to controls, with the highest and most consistent upregulation in the IC1‐GOM group, whereas LOH samples showed a more moderate increase (Figure S3B). *SIX1*, crucial in early kidney development, was the most significantly upregulated gene. Conversely, the kidney‐specific *UMOD* gene was most significantly downregulated. Although olfactory receptor genes were represented among differentially methylated loci, RNA‐seq coverage for these genes was extremely low in both tumour and control tissues, suggesting limited biological relevance in WT. Upregulated and downregulated DEGs were used for KEGG enrichment analysis. The top 10 enriched pathways are shown in Figure S3C,D, with scores and significance in Table S8. Upregulated pathways were mainly related to cancer processes (cell cycle, DNA replication, cell adhesion molecules), while downregulated pathways included kidney functions (proximal tubule bicarbonate reclamation) and cell proliferation or apoptosis (calcium signalling). Figure S3E illustrates the unsupervised hierarchical clustering of DEGs. PERMANOVA and dispersion tests found significantly different expression patterns in clusters of regressive and epithelial tumours, with an adjusted *p* value of 0.03696 for both (see Table S5).

Together, these findings suggest a distinct transcriptomic landscape in WT, characterised by activation of oncogenic processes and loss of kidney‐specific functions as well as association with histological diversity.

### 3.6 Integration of a Larger Transcriptomic Dataset Improved Stratification Into Molecular WT Subtypes

A limitation of our transcriptomic analysis is the small sample size, which hindered stratifying WT into molecular subgroups. Therefore, we included a filtered transcriptomic dataset of 102 WT samples from the St. Jude Cloud paediatric cancer data center (Table S9) [33]. Within this large reference dataset, we identified transcriptomic subtypes and their corresponding signature genes by Non‐negative Matrix Factorization that grouped tumours with similar gene expression patterns. We initially performed a rank survey analysis to find the optimal group number in the St. Jude Cloud dataset (Figure S4A). Rank 3 was selected as the optimal solution because it represented the best compromise between cluster stability, compactness and biological interpretability across cophenetic, dispersion and silhouette metrics. Consistently, PCA of rank‐3 metagene coefficients revealed three robust transcriptional clusters (Figure S4B). For each of these, we defined a set of genes that are both highly expressed and specific to that group using Kim's method, thereby generating subtype‐specific gene signatures. These genes were then used to develop a gene template that effectively discriminated the three WT subtypes by unsupervised hierarchical clustering (Table S10, Figure S4C).

We then used this gene template to analyse our cohort by Nearest Template Prediction analysis and confirmed three gene expression Subtypes 1–3 (Figure 4A). We observed a significant overlap between these groups and histological types (Fisher's test adjusted *p* value = 0.02296125, Table S5). In particular, Subtype 2 was enriched with stromal‐predominant tumours, while Subtype 1 mostly matched regressive samples and epithelial tumours with minimal alterations, including WT25 and WT27. Conversely, Subtype 3 was enriched in blastemal and mixed histological types, consistent with a less differentiated tumour composition. Interestingly, when we cross‐referenced these subtypes with methylation clusters, we observed a significant concordance (Fisher's test adjusted *p* value = 0.02296125), indicating consistency between gene expression and epigenetic stratification (Figure S5). The tumour microenvironment, a complex ecosystem of cancer and non‐cancerous cells (such as stromal and immune cells), extracellular matrix and signalling molecules, is known to influence tumour behaviour and contribute to cancer heterogeneity [34]. We studied the tumour microenvironment of our WTs using the ESTIMATE method, which predicts stromal and immune cell content and tumour purity from gene expression data. The analysis revealed significant differences among the three subtypes (Figure 4B). While Subtypes 1 and 2 showed higher stromal scores, with Subtype 1 also displaying higher immune infiltration, Subtype 3 had greater tumour purity, according to computational estimation [35]. Gene Set Variation Analysis revealed each group's pathways (Figure 4C). Subtype 1 (regressive enriched) strongly activated immune pathways like ‘JAK–STAT signalling’ and ‘T‐cell receptor signalling’. Subtype 2 (stromal predominant) up‐regulated metabolic and developmental pathways such as ‘Valine, leucine and isoleucine biosynthesis’ and ‘mTOR signalling’. Subtype 3 displayed changes in pathways affecting cell cycle and genome stability. As stromal‐predominant WTs are frequently associated with genetic alterations in *WT1* and *CTNNB1* [13, 36], we examined their expression across transcriptional subtypes and methylation clusters, with particular focus on Subtype 2 and Cluster 3. Interestingly, neither *WT1* nor *CTNNB1* expression differed significantly in these groups compared to the others. In contrast, *WT1* expression was significantly elevated only in Cluster 2 and Subtype 3 (Figure S6A,B), whereas *CTNNB1* expression was significantly increased across all methylation clusters and transcriptional subtypes compared to controls (Figure S6C,D).

**FIGURE 4:**
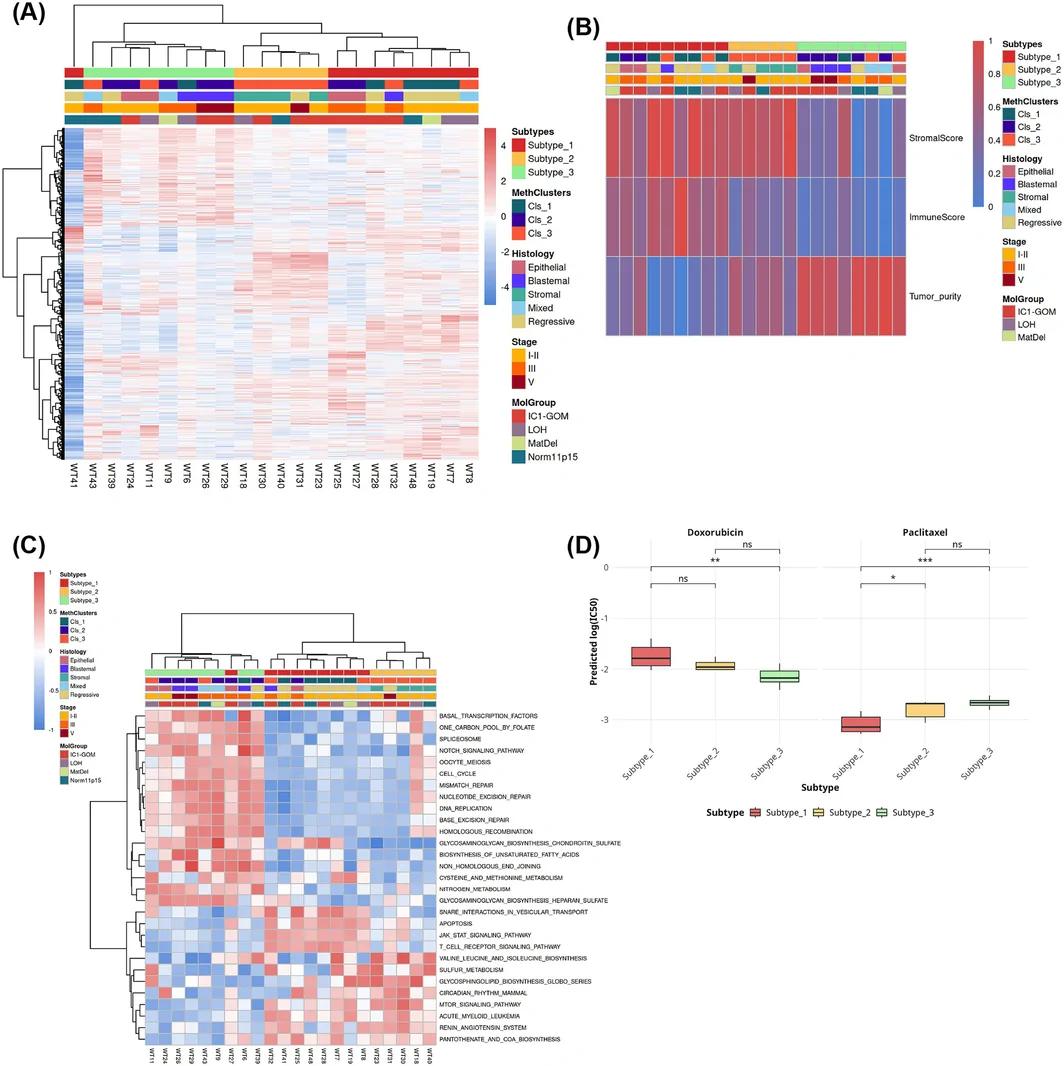
Expression‐based WT subtypes profiling. (A) Heatmap displaying the hierarchical clustering of NTP template genes in our WT samples successfully recapitulates three molecular subtypes. (B) Heatmap showing tumour microenvironment composition per sample as computed by the ESTIMATE algorithm. (C) Heatmap displaying GSVA enrichment scores for the most variable KEGG pathways across WT subtypes. (D) Boxplots showing predicted drug sensitivity (log(IC50) values) across WT subtypes for common chemotherapeutic agents. Lower log(IC50) values suggest higher predicted sensitivity. Asterisks indicate statistical significance between groups (Wilcoxon): Adjusted *p* value < 0.05 (*), adjusted *p* value < 0.01 (**), adjusted *p* value < 0.001 (***), ‘ns’ denotes non‐significant differences.

To predict possible subtype‐specific therapies in WT, we performed an in silico drug sensitivity analysis with standard chemotherapeutics applied to gene expression data. The results indicated subtype‐specific treatment effectiveness. While Subtype 3 was predicted to have greater sensitivity to Doxorubicin (median log(IC50) = −2.17) with respect to the other groups, Subtype 1 had a significantly higher sensitivity to Paclitaxel (median log(IC50) = −3.14), while intermediate sensitivity to both drugs was predicted for Subtype 2 (Figure 4D).

In summary, transcriptomic analysis of WT enabled the stratification into three tumour subtypes that correlated with clinical features and methylation profiles, providing therapeutic information for WT treatment.

### 3.7 Integrated Analysis Defined a Subset of Genes Controlled by Promoter Methylation in WT

To assess DNA methylation's impact on gene expression in WT, we intersected DEGs and promoter methylation, considering mean methylation of regions with ≥ 3 differentially methylated CpGs and *p* value < 0.05. We identified 564 hypomethylated and 188 hypermethylated promoters in upregulated DEGs, and 487 hypomethylated and 820 hypermethylated promoters in downregulated DEGs (Figure S7A). A Spearman correlation analysis identified genes significantly correlated with promoter methylation (|Rs| > 0.45 and an adjusted *p* value < 0.05). Most DEGs (931, 84%) showed negative correlation (ncDEGs), while only 171 (16%) were positively correlated (Figure S7B, Table S11). ncDEGs were either upregulated‐hypomethylated (376 genes, upper‐left corner) or downregulated‐hypermethylated (555 genes, lower‐left corner). Gene expression and promoter methylation of ncDEGs are compared in the heatmap of Figure S7C. Notably, unsupervised clustering of the ncDEGs reproduced the three expression‐based subtypes described above, and sample group discrimination tests yielded significant results for all three subtypes (Table S5), demonstrating that ncDEGs are sufficient to differentiate them.

KEGG analysis showed upregulated DEGs with promoter hypomethylation enriched in cancer‐related genes, especially in signalling pathways like Wnt and PI3K‐Akt, and immune markers such as cell adhesion molecules, immunoglobulin receptors and integrins (Figure S7D, Table S12). Conversely, the downregulated DEGs with hypermethylated promoters were mainly linked to metabolic pathways and tight junctions (Figure S7E, Table S12). The intersection of ncDEGs with the COSMIC cancer dataset (as accessed March 13, 2025) identified 43 genes (Table S13). Consistent with their down‐regulation, we found increased promoter methylation of many tumour suppressors (approximately 53% of down‐hyper genes), such as *SDHB* and *CDH1*. Conversely, promoter methylation of upregulated oncogenes (60% of up‐hypo genes), such as *FGFR1* and *CD28*, was decreased. Differently, the promoter of the downregulated kidney epithelium marker *PAX8* was found to be hypermethylated. Finally, in line with the hypomethylation of its promoter and the global hypomethylation of the genome, we observed upregulation of *TET1*, a gene involved in active DNA demethylation. About half of these genes (23/43) were associated with specific subtypes, mostly Subtype 1 (e.g., *CDH1*, *LRP1B*, *CD28*, *CSF1R*) and Subtype 3 (e.g., *PAX8*, *AFF3*, *BRCA1*, *CNTNAP2*). Some scatter plots of the correlation between promoter methylation and gene expression are shown in Figure S8.

In summary, we identified many deregulated genes controlled by promoter methylation in WT, including tumour suppressors and oncogenes characterising WT subtypes.

### 3.8 Mechanisms Underlying the Dysregulation of the *IGF2/H19* Locus in WT

Given the high incidence of *IGF2* and *H19* expression changes in WT, we investigated the underlying molecular mechanisms. The methylation status of IC1, altered in both IC1‐GOM and LOH groups (78% of samples), influences and creates hierarchical DNA methylation changes nearby [37]. Consistent with these observations, *H19* suppression correlated with promoter hypermethylation in these cases (Table S11). Conversely, increased expression of *IGF2* was not associated with hypomethylation of its annotated P0 promoter, but rather with IC1 hypermethylation (Figures 5A,B and S9A). However, activation of *IGF2* may also be triggered by alternative promoters in cancer [18]. To clarify this point, we examined the methylation status of individual *IGF2* promoters (P0–P4) and corresponding RNA isoform expression (Figure 5A–C). Excluding the liver‐specific P1, we found that while P0 remained normally methylated, P2–P4 showed significant hypomethylation in tumours compared to controls, although P3 and P4 were normally methylated at a very low level (Figure 5B). Notably, all *IGF2* isoforms were upregulated in tumours, especially from P3 and P4 (Figure 5C). Quantile regression showed a positive correlation between IC1 methylation and *IGF2* expression at low activation levels (10–30th percentile). Among promoters, only P4 had a modest negative effect around median quantiles (Figure S9C, Table S5). Regarding secondary *IGF2* DMRs, DMR2 methylation remained unaffected, but DMR0 was hypomethylated (Figure 5B) and inversely correlated with *IGF2* expression in WTs versus NNKs (Figure S9B). Notably, DMR0 methylation was negatively associated with *IGF2* expression, especially in upper quantiles (median to 90th percentile) (Figure S9C, Table S5).

**FIGURE 5:**
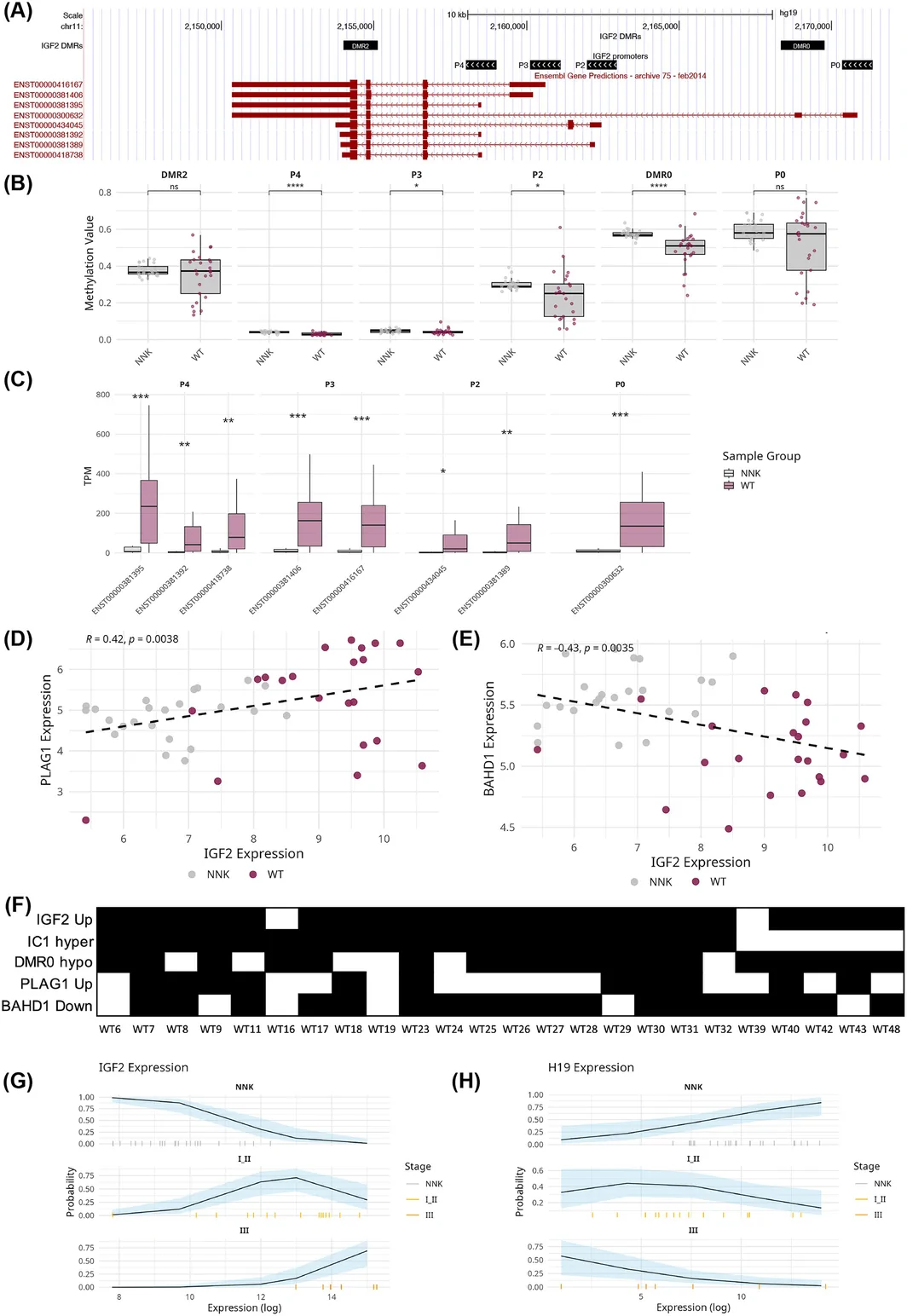
Molecular alterations in the *IGF2/H19* locus and correlation with defects in regulatory factors. (A) Screenshot of the *IGF2* locus from the UCSC Genome Browser showing imprinted DMRs, promoters and transcript isoforms. (B) Boxplots showing DNA methylation across key regulatory elements of the *IGF2* locus. Each dot represents the mean beta values for each WT (pink) and NNKs (grey) sample. Statistical significance is indicated by asterisks (Paired Wilcoxon): Adjusted *p* value < 0.05 (*), < 0.01 (**), < 0.001 (***) and < 0.0001 (****); ‘ns’ denotes non‐significant differences. (C) Boxplots showing the expression levels of *IGF2* transcript isoforms driven by distinct promoters. Statistical significance between WT and NNK is indicated by asterisks (Paired Wilcoxon): Adjusted *p* value < 0.05 (*), < 0.01 (**), < 0.001 (***) and < 0.0001 (****). Scatter plot showing the positive (D) and negative (E) correlation between *PLAG1* and *IGF2* expression levels and between *BAHD1* and *IGF2* expression levels, respectively. The dotted line represents a linear regression fit (lm) that indicates the direction and strength of correlation. (F) Schematic representation of molecular defects across all WT samples for which methylomic and transcriptomic analyses have been performed, showing the presence (black) or absence (white) of specific molecular alterations. Rows represent key regulatory defects: *IGF2* Up (upregulation, 0.5logFC), IC1 hyper (hypermethylation, ≥ 2.5 SD), DMR0 hypo (hypomethylation, ≤ 2.5 SD), *PLAG1* Up (upregulation, 0.5logFC), and *BAHD1* down (downregulation, 0.5logFC). Columns correspond to individual WT samples. Line plots depicting the results of ordinal regression analyses modelling the probability of tumour stage as a function of log‐transformed *IGF2* (G) and *H19* (H) expression. Each plot is divided into three vertically stacked facets representing distinct stages: NNK, Stage I–II, and Stage III WTs. The x‐axis shows log‐transformed expression values, while the y‐axis represents the predicted probability of belonging to a given stage. The black line in each facet shows the estimated probability of that stage across the expression spectrum, and the blue ribbon represents the 95% confidence interval around the estimate. A coloured rug plot at the bottom of each facet indicates the distribution of samples belonging to the respective stage.

We then searched for trans‐acting regulatory factors by analysing genes outside the *IGF2/H19* locus whose expression correlated with *IGF2* levels across samples. Among the identified candidates, *PLAG1*, a known activator of *IGF2* transcription [38, 39], was consistently upregulated in tumours and showed a significant positive correlation with *IGF2* expression (*R* = 0.42, *p* = 0.0038; Figure 5D). In contrast, *BAHD1*, a chromatin‐associated repressor [40], showed a significant negative correlation with *IGF2* levels (*R* = −0.43, *p* = 0.0035; Figure 5E). A linear regression analysis further supported these associations, showing a positive effect of *PLAG1* (0.6282, *p* = 0.0038), and a negative effect of *BAHD1* on *IGF2* expression (−1.7901, *p* = 0.0027). The model explained about 30% of *IGF2* variability and was statistically significant. To assess whether *PLAG1* and *BAHD1* jointly regulate *IGF2* or act independently, we split tumour samples into two groups: high‐*PLAG1* (above median NNKs) and low‐*PLAG1* (below median). In the high‐*PLAG1* samples, no significant link between *IGF2* and *BAHD1* levels (*p* = 0.1021) was found suggesting *PLAG1* regulation in this group (see Table S5). In the low‐PLAG1 group, *IGF2* and *BAHD1* showed a significant negative correlation (*p* = 0.03505), indicating that *BAHD1* may regulate *IGF2* more effectively in the absence of *PLAG1* overexpression. Figure 5F summarises molecular changes linked to *IGF2* activation in our WT cohort.

Finally, we studied *IGF2* and *H19* expression during tumour progression using ordinal regression analysis. Results showed different behaviours (Figure 5G,H, Table S5). *IGF2* levels increased progressively from controls to Stages I–II (*p* value 1.24 × 10^−4^), with a further rise in Stage III (*p* value 6.25 × 10^−5^). Conversely, *H19* decreased from controls to Stage I–II tumours (*p* value 1.310^−2^), with no significant change from Stage I–II to III (*p* value 0.50).

In summary, these results suggest that *H19* downregulation marks early WT tumourigenesis, likely due to IC1 hypermethylation. *IGF2* activation, occurring at all stages regardless of IC1 hypermethylation, is probably initially triggered by this defect and subsequently influenced by additional *cis*‐acting alterations within the *IGF2/H19* locus (including DMR0 and promoter usage) together with *trans*‐acting regulatory factors such as PLAG1 and BAHD1.

## 4 Discussion

Epigenetic defects in the 11p15.5 region are common in WT [6]. We confirmed that over 70% of WTs have abnormalities, especially IC1‐GOM and copy‐neutral LOH, emphasising the key role of the *IGF2/H19* locus in WT development. Our genome methylation analysis also found additional DNA methylation changes beyond the 11p15.5 locus, including widespread intergenic hypomethylation and promoter methylation alterations, revealing a new epigenomic WT footprint that affects many cancer genes. This epigenetic signature is highly specific compared with control tissue, and with methylomes from publicly available normal foetal kidney and other infant solid tumours. Currently, since biopsy is not routinely recommended, diagnosis is confirmed by histological analysis after surgery, which also determines the risk grade [3]. However, this protocol may be limited by difficulties in distinguishing WT from other abdominal masses, such as neuroblastoma, or in diagnosing very young children or extrarenal WT cases. The epigenetic signature may therefore represent a novel diagnostic tool, as a highly sensitive and specific molecular marker for all difficult‐to‐diagnose cases. Moreover, to monitor treatment response or identify relapse in patients already diagnosed, as well as to early detect the onset in cases with predisposing syndromes, this WT episignature represents a novel molecular biomarker worth testing for the detection of circulating tumour DNA.

Our methylation clustering revealed three epigenetic WT groups significantly linked to histological categories. One cluster, mostly regressive tumours, showed minimal epigenetic disruption and resembled non‐neoplastic tissue. Regressive WTs undergo major changes post‐chemotherapy, especially necrosis [41]. Therefore, the mild epigenetic dysregulation probably indicates fewer viable tumour cells rather than a different molecular profile. However, we cannot exclude the possibility that regressive‐type WTs exhibit fewer initial epigenetic changes, which may explain their better response to chemotherapy. Indeed, since epigenetic modifications can both promote tumour growth and confer resistance to chemotherapy [42], WTs with fewer epigenetic changes might be more susceptible to chemotherapy's cell‐killing effects. From this perspective, it is noteworthy that regressive WTs mostly lacked alterations in the 11p15.5 region. Since all tumours were collected after preoperative chemotherapy, treatment‐related epigenetic effects cannot be excluded. Analysing epigenetic profiles before and after chemotherapy will help clarify the relationship between WT epigenetics and treatment effects.

A previous report found consistent and pronounced hypomethylation in satellite regions, corresponding to repetitive pericentromeric heterochromatin, in IC1‐GOM tumours compared to LOH and Norm11p15.5 cases [31]. Although repetitive satellite regions are largely excluded from the design of the Illumina EPIC array used in our study and were therefore not directly analysed in our dataset, our findings confirmed subgroup differences and support the existence of widespread epigenetic dysregulation in IC1‐GOM cases, rather than isolated IC1 hypermethylation. We found that both hypomethylation and hypermethylation characterise the epigenetic disturbances in this group, affecting both intergenic regions and genes, particularly those disrupting cellular environment and promoting tumour development. These findings are consistent with the notion that IC1‐GOM represents an early predisposing epigenetic event [12, 13, 43, 44], whereas the widespread epigenetic alterations observed in these tumours are likely acquired during tumour progression. The pronounced dysregulation of DNA methylation regulators suggests that IC1‐GOM tumours may be intrinsically more susceptible to epigenetic instability, leading to a broader reprogramming of the methylome beyond the 11p15.5 locus. This supports the higher WT risk reported in BWSp patients with IC1‐GOM, which is approximately threefold higher than in pUPD11/LOH cases and sixfold higher than in BWSp patients without detectable 11p15.5 alterations, corresponding to the Norm11p15.5 subgroup in our cohort [45]. Moreover, mosaic ICR1 hypermethylation has been reported in nearly 60% of bilateral WT cases [43]. Consistent with this observation, two of the four cases in our cohort showing IC1‐GOM in NNK tissue were bilateral WTs. We also found that epigenetic defects in LOH and Norm11p15.5 samples were more restricted and heterogeneous, indicating genetic rather than epigenetic abnormalities. Indeed, LOH is a somatic recombination process contributing to genomic instability and cancer that acts as a ‘second hit’ by causing loss of the wild‐type allele of tumour suppressor genes already inactivated by earlier events [46]. In WT, for instance, LOH involving both the 11p15.5 and *WT1*/11p13 loci is often associated with *WT1* variants [13]. As suggested by previous reports, imprinting dysregulation beyond the 11p15.5 locus emerges as a characteristic feature of WT [23, 47]. It appears to be largely tumour‐associated rather than subgroup‐defining, with only limited involvement of a few loci in control samples with IC1‐GOM predisposition. Notably, the extensive dysregulation of imprinted gene expression is only partially explained by alterations in the methylation of the corresponding DMRs, indicating that additional mechanisms are likely to contribute in WT.

The transcriptomic analysis of WTs revealed a gene expression profile marked by activated cancer pathways and suppressed kidney‐specific genes. These confirmed previous findings and highlight WT's dual nature as both a developmental and a cancerous disorder, with oncogenic processes alongside loss of lineage fidelity [11]. Comparing our cohort with the large St. Jude Cloud transcriptomic dataset, we stratified WTs into three molecular subtypes reflecting histological composition and confirming disease heterogeneity [48]. Subtype 1 was enriched in regressive tumours and characterised by an immune‐related microenvironment and inflammatory pathways, suggesting immune‐driven regression. In line with this, a more inflamed WT microenvironment with higher immune infiltration correlates with better outcomes [49]. However, an intrinsic tumour‐immune interaction contributing to the more favourable behaviour of this subtype cannot be excluded. In contrast, Subtype 3 showed higher tumour purity and deregulation of pathways involved in cell cycle and genomic stability, indicating a more aggressive, proliferative phenotype. High‐risk blastemal‐type and advanced‐stage WTs were enriched in Subtype 3. Finally, the involvement of metabolic and developmental pathways in Subtype 2 suggests a mesenchymal‐like reprogramming typical of stromal‐type WTs [50]. Due to the limited number of bilateral or bifocal cases in our cohort, no statistically significant association with methylation clusters or transcriptomic subtypes could be established. However, unlike previous observations [20], none of these cases clustered within Subtype 1, the subgroup associated with more favourable biological features. Notably, the pronounced correlation between transcriptomic subtypes and DNA methylation clusters indicates that methylation patterns shape transcriptional programmes responsible for tumour identity, a finding increasingly recognised in embryonal tumours [51]. In this context, our findings extend the molecular stratification previously proposed [20], demonstrating that combining methylation and transcriptional data offers a more detailed understanding of prognostically relevant WT subgroups. *WT1* overexpression in WT has been linked to maintenance of epithelial‐mesenchymal balance and proliferative blastemal states, with a proposed oncogenic role [52]. Its higher expression in Cluster 2/Subtype 3, characterised by high tumour purity and low immune infiltration, further supports its association with aggressive tumour features. In parallel, *CTNNB1* expression was consistently elevated across all molecular groups, in line with the well‐established activation of the Wnt/β‐catenin pathway in WT [53]. We defined a subset of DEGs regulated by promoter methylation that reproduced the three molecular subtypes, offering potential biomarkers for stratification and epigenetic therapy targets, and emphasising the need for further validation in larger cohorts. The in silico chemosensitivity analysis provided insights into postoperative treatments, predicting subtype‐specific responses and proposing molecular stratification for precision therapy in WT. Doxorubicin causes DNA damage, leading to cell cycle arrest and apoptosis, and is advised for higher‐risk WTs [4]. Our data support this idea and, based on molecular changes in Subtype 3 WTs, predict more suitable cases. Subtype 1 WTs might benefit from Paclitaxel, which has recently been suggested to boost immune cell activity [54, 55, 56, 57]. Although these predictions need experimental validation, they show the potential of integrative omics to guide personalised WT oncology treatment.

Our analyses of DNA methylation and gene expression reveal new insights into *IGF2/H19* locus dysregulation in WT. *IGF2* upregulation likely depends on IC1 hypermethylation, either through loss of imprinting in the IC1‐GOM subgroup or double expression from paternal alleles in the LOH subgroup [14]. Indeed, our results indicated that IC1 hypermethylation contributed to a relatively low upregulation of *IGF2*. This suggests that stronger *IGF2* upregulation in WT occurs via other molecular mechanisms. Promoter activation could be one of these, as we observed upregulation of all *IGF2* isoforms. Moreover, *IGF2* expression levels significantly correlated with *PLAG1* and *BAHD1* expression. PLAG1 is a transcription factor suggested to play a role in WT, as it binds the P3 promoter in Wit49 cells and increases *IGF2* levels in various tumours [38, 39, 58]. A novel finding of our study is the inverse correlation between *IGF2* and *BAHD1*, a gene encoding an H3K27me3 reader with repressor activity [40]. Interestingly, in HEK293 cells, BAHD1 colocalised with H3K27me3, contributing to the repression of PRC2 target genes, including *IGF2* [59, 60]. In particular, it was shown to interact with an extended region from DMR0 to P4, with a stronger binding on P3. These findings together suggest that the chromatin state of *IGF2* regulatory regions, rather than modest changes in promoter DNA methylation alone, plays a key role in its activation in WT. In particular, the repressive histone mark H3K27me3, recognised by BAHD1, appears to play a key role in maintaining *IGF2* silencing. We therefore propose a model in which BAHD1 sustains physiological repression of *IGF2* in NNK, whereas in WT reduced *BAHD1* expression may lead to derepression of *IGF2* promoters, with additional contribution from PLAG1‐mediated transcriptional activation. This dual regulatory axis may also explain *IGF2* activation in WTs without IC1 methylation defects. We previously found DMR0 demethylation in WT cases with IC1 hypermethylation, either IC1‐GOM or LOH [61]. Here, we show that this alteration also occurs in WTs with normal methylation and is strongly associated with *IGF2* overexpression, especially at higher levels. DMR0 has been proposed as a P0‐specific enhancer, whose activity is regulated by DNA methylation in Sotos syndrome [62]. Our data suggest that reduced DMR0 methylation also enhances *IGF2* expression from the other more active promoters in WT. In an old report, biallelic *IGF2* activation was associated with WT progression [63]. Here, we observed early *H19* suppression at tumour onset, while *IGF2* activation increased with progression, suggesting a model where initial imprinting defects lead to tumour initiation, followed by enhanced *IGF2* activation during tumour expansion. These findings support a multilayered regulatory model where IC1 hypermethylation primarily causes imprinting relaxation, while stronger *IGF2* overexpression during tumour progression appears to be additionally influenced by other *cis*‐acting alterations identified within the locus (including DMR0 hypomethylation and altered P3‐P4 promoter activity) together with *trans*‐acting factors identified through expression‐correlation analyses (PLAG1, BAHD1). Understanding these mechanisms may help develop targeted epigenetic or transcriptional therapies to reduce *IGF2*‐driven oncogenic signalling in WT.

In conclusion, this study presents a comprehensive model of epigenetic deregulation in WT, revealing insights into tumour heterogeneity, DNA methylation‐driven gene regulation and *IGF2* dysregulation. However, several limitations should be acknowledged. First, the cohort size, while enough to identify major trends, is relatively small, and validation with larger cohorts will be vital for definitive prognostic stratification. Second, although drug sensitivities were inferred from gene expression profiles, these predictions need experimental validation. Prospective preclinical testing in patient‐derived organoids or xenograft models is crucial to evaluate subtype‐specific responses and determine the most promising therapies. Lastly, while our study indicates regulatory roles for PLAG1 and BAHD1 in *IGF2* expression, functional validation through in vitro and in vivo experiments is necessary to understand their molecular mechanisms and potential as therapeutic targets. Despite these limitations, our findings offer new biomarkers and potential therapeutic targets, supporting future improvements in molecular classification and personalised treatment strategies in WT.

## Author Contributions

**Abu Saadat:** conceptualization, investigation, writing – original draft, methodology, software, data curation. **Francesco Cecere:** conceptualization, investigation, data curation, methodology, software. **Federica Rossetti:** methodology, investigation, software. **Laura Pignata:** methodology. **Emilia D'Angelo:** methodology. **Carlo Giaccari:** methodology. **Gaetano Verde:** resources. **Paola Quarello:** resources. **Franca Fagioli:** resources. **Josep Roma:** resources. **Manel Esteller:** supervision. **Enrique de Álava:** resources. **Flavia Cerrato:** writing – review and editing, funding acquisition. **Andrea Riccio:** supervision, conceptualization, investigation, funding acquisition, writing – review and editing. **Angela Sparago:** conceptualization, investigation, funding acquisition, writing – original draft, supervision, writing – review and editing.

## Funding

This work was supported by: Associazione Italiana per la Ricerca sul Cancro, IG 2020 ID 24405; Ministero dell'Università e della Ricerca, PRIN 2022B2N2BY; Accordo per la Coesione della Regione Campania (Project MARADONA).

## Ethics Statement

This study was performed in accordance with the Declaration of Helsinki. Samples and related information were collected with approval from the ethical committees of the University of Campania ‘Luigi Vanvitelli’ (Prot. 0010423/i‐05/05/2020), Hospital Universitario ‘Virgen del Rocío’ (52a21bdb0fe951adc9ea75883b032a4d40d37033–15/05/2020), and Azienda Ospedaliero Universitaria Città della Salute e della Scienza di Torino (MTA approval of the Nefro‐GenFen study–18/11/2021). All participants provided informed consent for their tissue samples to be used in research. All data are shown as deidentified samples.

## Conflicts of Interest

The authors declare no conflicts of interest.

## Supporting information


**Table S1:** IC1 and IC2 DNA methylation levels.
**Table S2:** RNAseq details.
**Table S3:** List of Differentially Methylated CpG Sites (DMSs).
**Table S4:** KEGG enrichment of DMSs.
**Table S5:** Statistic analyses.
**Table S6:** KEGG enrichment of 11p15.5 molecular subgroups‐specific DMSs.
**Table S7:** List of Differentially Expressed Genes (DEGs).
**Table S8:** KEGG enrichment of DEGs.
**Table S9:** Sample Info of St. Jude cohort.
**Table S10:** Subtype template.
**Table S11:** Correlation between gene‐expression and promoter DNA methylation.
**Table S12:** KEGG enrich of negatively correlated DEGs.
**Table S13:** COSMIC negatively correlated DEGs.

**Figure S1:** Comparison of the global DNA methylation levels of WT and control. (A) Violin plots showing the distribution of methylation beta values across all CpG sites profiled by the Illumina EPIC array in WT (pink) and NNK (grey) samples. The y‐axis represents beta values, reflecting methylation levels from 0 (unmethylated) to 1 (fully methylated). Asterisks indicate statistical significance between groups (Paired Wilcoxon): **** denotes *p* < 0.0001. (B) Heatmap showing the hierarchical clustering of DMSs associated with genes belonging to pathways in cancer. (C) Principal component analysis of genome‐wide DNA methylation profiles in NNK samples. Percentages indicate the variance explained by each principal component.
**Figure S2:** Imprinted methylation and gene expression alterations across 11p15.5 molecular subgroups of WT. Heatmaps showing DNA methylation levels at imprinted differentially methylated regions (DMRs) (A) and expression profiles of imprinted genes (B) across control samples and 11p15.5 subgroups of WTs. Heatmaps represent scaled methylation or expression values, with red indicating higher and blue indicating lower relative levels. Samples from cases with IC1‐GOM predisposition are circled in green.
**Figure S3:** Gene expression and pathway alterations in WT. (A) Volcano plot of differentially expressed genes (DEGs) between WTs and NNKs. Genes meeting both statistical and biological thresholds (adjusted *p* value < 0.05 and |log2FC| ≥ 1) are shown in red. Genes with significant adjusted *p* values but lower fold changes are shown in blue, while genes with large fold changes (|log2FC| ≥ 1) but without statistical significance are shown in green. Genes not meeting either threshold are shown in grey. (B) Boxplots showing log2‐normalised IGF2 expression across 11p15.5 molecular subgroups. Statistical significance is indicated as follows: *p < 0.05; **p < 0.01; ****p < 0.0001. Dot plot showing KEGG pathway enrichment of genes upregulated (C) and downregulated (D), respectively. Dot colour reflects adjusted *p* value from enrichment analysis (red = higher significance, blue = lower significance), and dot size represents recall, defined as the proportion of genes from a given pathway that are present in the input gene set. (E) Heatmap showing the hierarchical clustering of 4,673 DEGs. Each column represents a sample, and each row a gene.
**Figure S4:** Rank survey analysis and subtype‐specific gene template derived from the St. Jude WT cohort's expression data. (A) Multi‐facet line plot depicting cophenetic coefficient, dispersion and average silhouette respectively. The metrics (y‐axis) were calculated for each tested factorization rank (x‐axis) NMF consensus clustering. Higher cophenetic and silhouette values together with lower dispersion indicate more stable and well‐separated cluster structures. (B) PCA plot of the coefficient matrix obtained from NMF with rank 3. Each point represents a sample. The separation of points along principal components reflects the distinct cluster assignments derived from the factorization. (C) Heatmap showing the top contributing genes for each of the three subtypes.
**Figure S5:** Correlation between gene‐expression subtypes and DNA methylation clusters. UpSet plot showing overlap between gene‐expression WT subtypes and methylation clusters (21 WT samples, Fisher's exact test adjusted *p* value = 0.02296125). The horizontal bar chart (left) shows the sizes of each individual set, while the vertical bar chart (top) displays intersection sizes, indicating the number of samples belonging to specific combinations of subtypes and methylation clusters. Connected dots in the matrix (bottom) represent which sets are included in each intersection.
**Figure S6:** WT1 and CTNNB1 expression across methylation‐based clusters and transcriptomic subgroups. (A) WT1 expression across methylation clusters (Cls_1‐Cls_3) and (B) transcriptomic subtypes (Subtype_1‐Subtype_3). (C) CTNNB1 expression across methylation clusters (Cls_1‐Cls_3) and (D) transcriptomic subtypes (Subtype_1‐Subtype_3). Statistical significance is indicated as follows: ns, not significant; *p < 0.05; **p < 0.01; ***p < 0.001.
**Figure S7:** Integrative analysis of DNA methylation and gene expression. (A) Venn diagrams showing the overlap between upregulated DEGs (top) and downregulated DEGs (bottom) with promoter methylation. A gene was considered to be differentially methylated if there was at least three DMSs in its promoter region (5′UTR, TSS1500, TSS200 and 1st Exon). (B) Scatter plot showing the correlation between promoter methylation and gene expression for informative DEGs. The x‐axis represents the Spearman correlation coefficient, and the y‐axis represents the log2 fold change in gene expression. Each point represents a gene; dot colour reflects the adjusted *p* value of the methylation‐expression correlation (red = more significant, blue = less significant). Vertical dashed lines indicate a correlation threshold of ±0.45. (C) Hierarchical clustering of 931 DEGs with significant inverse correlation between gene expression and promoter methylation (left panel) and corresponding promoter methylation levels for the same set of genes (right panel). The clustering order from the expression heatmap is preserved in the methylation heatmap to allow direct visual comparison. Dot plot showing KEGG pathway enrichment of genes significantly upregulated by promoter hypomethylation (D) and significantly downregulated by promoter hypermethylation (E), respectively. The dot colour and size indicate the adjusted *p* value and recall, respectively.
**Figure S8:** Examples of correlations between cancer gene‐expression and promoter DNA methylation. Scatter plots depicting gene‐expression and promoter DNA methylation levels for some ncDEGs.
**Figure S9:** Correlation of IGF2 expression with the DNA methylation of its regulatory regions. Scatter plots depicting the relationship between IGF2 expression (log‐transformed; x‐axis) and DNA methylation levels (b value; y‐axis) at two key regulatory regions: IC1 (A) and DMR0 (B). Each point represents an individual sample. (C) Multi‐facet line plots summarising quantile regression analysis of IGF2 expression versus methylation at five 11p15.5 loci (IC1, P4, P3, P2 and DMR0). The x‐axis shows the expression quantiles (τ = 0.1 – 0.9), and the y‐axis gives the regression coefficient for each quantile. Solid lines trace the estimated coefficients and the shaded ribbons denote 95% confidence intervals. Coefficients above zero indicate a positive association, while coefficients below zero indicate a negative association. Associations are significant where the confidence interval does not include the horizontal zero line.

## Data Availability

Methylomic and transcriptomic data were deposited into the GEO database with the accession numbers GSE303580 and GSE303967, respectively. Additional paediatric tumour DNA methylation datasets were downloaded from TARGET and Gene Expression Omnibus (GEO) databases (GSE69502; GSE132399; GSE131350). A gene‐expression dataset of additional WT samples was accessed from St. Jude Hospital databank. Further information is available from the corresponding authors upon request.
