# Supplementary material for: DNA Methylation and Transcriptomic Profiles of Wilms Tumour Reveal New Deregulated Genes and Epigenetic Processes Relevant for Tumour Stratification and Management
Source: Int J Cancer. 2026 Jul 30;159(11):2736–54. doi: 10.1002/ijc.70680 (PMC13637058; doi:10.1002/ijc.70680)
Supplement: Supplementary file 1 [file IJC-159-2736-s002.pdf]

DNA methylation and transcriptomic profiles of Wilms tumour reveal new deregulated genes and epigenetic processes relevant for tumour stratification and management

Abu Saadat
Francesco Cecere
Federica Rossetti
Laura Pignata,
Emilia D'Angelo
Carlo Giaccari
Gaetano Verde
Paola Quarello
Franca Fagioli
Josep Roma
Manel Esteller
Enrique De Álava
Flavia Cerrato
Andrea Riccio
Angela Sparago

Table of Content:

- Supplementary Methods
  - Bioinformatic and Statistical analysis
  - Processing of publicly available data

- Supplementary Tables (in a separate Excel file)
  - Table S1. IC1 and IC2 DNA methylation
  - Table S2. RNAseq details
  - Table S3. List of Differentially Methylated CpG Sites (DMSs)
  - Table S4. KEGG enrichment of DMSs
  - Table S5. Statistic analyses
  - Table S6. KEGG enrichment of 11p15.5 molecular subgroups-specific DMSs
  - Table S7. List of Differentially Expressed Genes (DEGs)
  - Table S8. KEGG enrichment of DEGs
  - Table S9. Sample Info of St. Jude cohort
  - Table S10. Subtype Template
  - Table S11. Correlation between gene-expression and promoter DNA methylation
  - Table S12. KEGG enrich of negatively correlated DEGs
  - Table S13. COSMIC negatively correlated DEGs

- Supplementary Figures
  - Figure S1. Comparison of the global DNA methylation levels of WT and control
  - Figure S2. Imprinted methylation and gene expression alterations across 11p15.5 molecular subgroups of WT
  - Figure S3. Gene expression and pathway alterations in WT
  - Figure S4. Rank survey analysis and subtype-specific gene template derived from the St. Jude WT cohort's expression data

- Figure S5. Correlation between gene-expression subtypes and DNA methylation clusters
- Figure S6. *WT1* and *CTNNB1* expression across methylation-based clusters and transcriptomic subgroups
- Figure S7. Integrative analysis of DNA methylation and gene expression
- Figure S8. Examples of correlations between cancer gene-expression and promoter DNA methylation
- Figure S9. Correlation of *IGF2* expression with the DNA methylation of its regulatory regions

- Supplementary References

SUPPLEMENTARY METHODS

Bioinformatic and Statistical analyses

All bioinformatic analyses were performed using the R statistical software (version 4.4.3) and Bioconductor (version 3.19.1). All results were visualised with ggplot2 (v3.5.1) and pheatmap (v1.0.12) R packages. Pair effects were controlled across all omics assays using mixed- or fixed-effect models as needed. For methylation array data, intra-pair correlation was calculated using 'duplicateCorrelation', and pairs were modeled as random effects via the block parameter in limma. For RNA-seq data, pair effects were modeled as fixed effects in the design matrix of the DESeq2 analysis. The dataset was balanced for gender, and all samples were of similar age; therefore, no additional correction for these variables was required. P-values were adjusted wherever relevant using Benjamini-Hochberg method and values below 0.05 were considered significant unless mentioned otherwise. Euclidian distance and 'Ward-D2' algorithm were used in all clustering analysis.

Differential methylation analysis was performed on M-values of tumours and controls using Limma (v3.60.6) package [1]. Geneset enrichment analysis was performed using rGREAT (v2.6.0) package [2]. The enriched terms were filtered for an adjusted hypergeometric p-value below 0.05. PERMANOVA (Permutational Multivariate Analysis of Variance) tests were performed using the adonis2 function from the vegan R package (v2.6-10). The significance of group separation was evaluated using 1,000 permutations. To confirm that the observed difference was not driven by unequal within-group dispersion, beta-dispersion was calculated, followed by ANOVA using the stats package (v4.4.3) in R to test for homogeneity of variances across groups.

The expression data were processed using CPM filtering implemented in NOIseq (v2.38.0) [3] with default parameters. The filtered data was normalised using upperquartile normalisation from the EDAseq package (v2.28.0) [4]. Differential expression analysis was performed using the DESeq2 R package (v1.44.0) [5]. KEGG pathway over-representation analysis was performed using gprofiler2 package (v0.2.3) [6]. Ordinal and quantile regressions were performed using MASS (v7.3-64) and quantreg (v5.99) R packages. All visualizations were based on z-scores or log transformed values.

Spearman correlation was computed between gene expression and mean promoter (5'UTR, TSS1500, TSS200, 1stExon) methylation levels, using the correlation R package (v0.8.6) and correlations were deemed significant if the Spearman coefficient exceeded 0.45 with an adjusted p-value < 0.05.

Non-negative Matrix Factorization (NMF) was performed using NMF R package (v0.28) [7]. The optimal number of clusters was determined using cophenetic, dispersion and silhouette statistics. Nearest Template Prediction (NTP) was used to assign subtypes in the study cohort based on correlation with expression signatures defined using Kim's method in 'extractFeatures' function. Overlap between expression subtypes and clinical information was evaluated using Fisher's exact test. To evaluate the tumour microenvironment, the ESTIMATE R package (v1.0.13) was used to infer the tumour purity, with KEGG pathway annotations obtained via the msigdbr package (v7.5.1). Drug sensitivity across subtypes was predicted for commonly used WT chemotherapeutics using the pRRophetic[8], with the "tissueType" parameter set to "allSolidTumors".

Processing of publicly available data

Datasets from TCGA were accessed and pre-processed using the TCGABiolinks package (v2.32.0) [9] with default parameters. GEO datasets were processed as described for the study cohort. The datasets were merged using common CpGs. Transcriptomic data for WT samples were obtained from the St. Jude Cloud Pediatric Cancer Data Center. Starting from the complete dataset, only primary tumour samples were retained, whereas xenograft-derived samples, relapse specimens, and germline samples were excluded. In addition, three samples (SJWLM034112_D1, SJWLM030043_D1, and SJWLM071563_D1) were identified as clear outliers by principal component analysis of the global expression matrix and removed prior to downstream analyses.

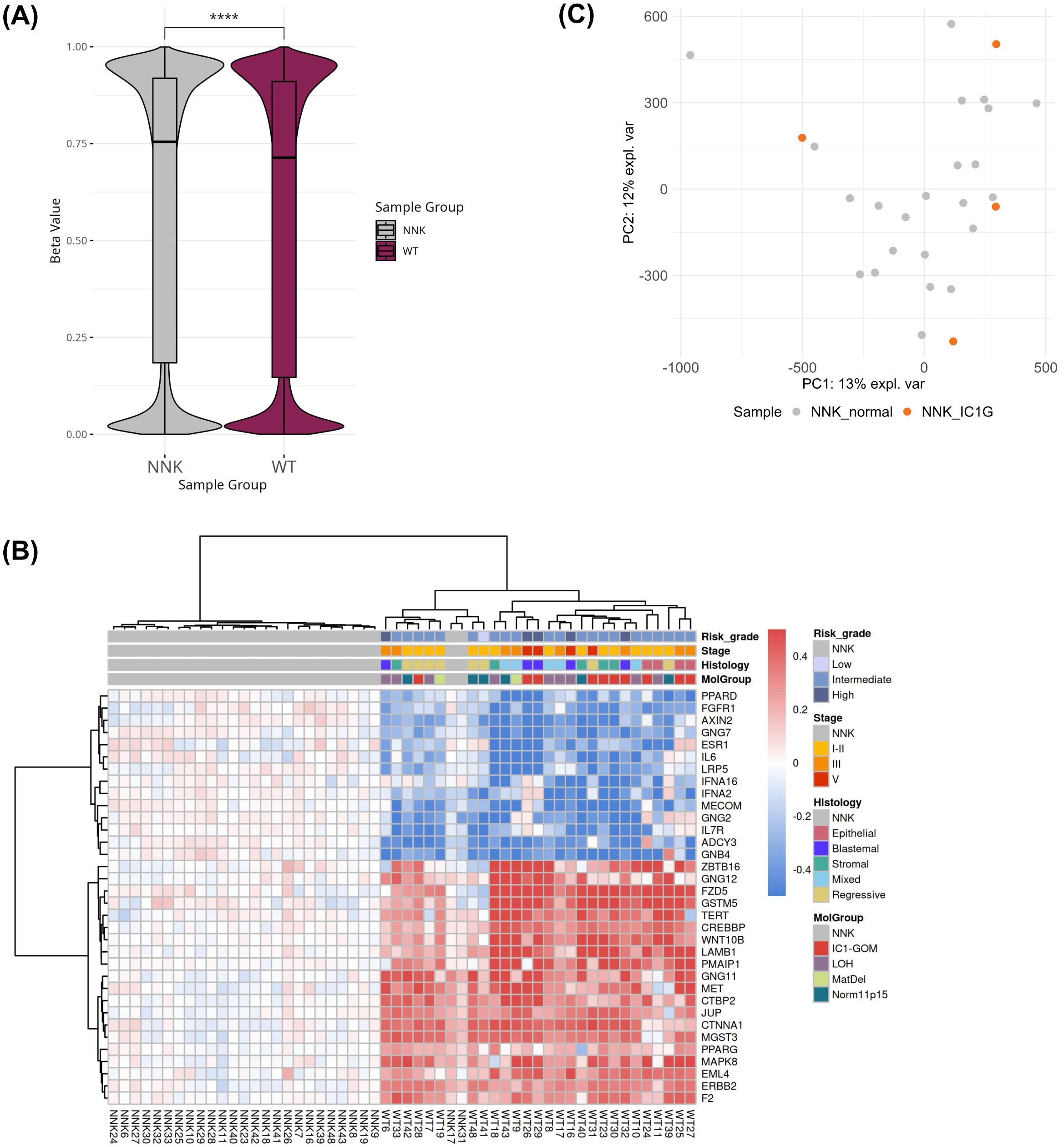

Figure S1. Comparison of the global DNA methylation levels of WT and control
(A) Violin plots showing the distribution of methylation beta values across all CpG sites profiled by the Illumina EPIC array in WT (pink) and NNK (grey) samples. The y-axis represents beta values, reflecting methylation levels from 0 (unmethylated) to 1 (fully methylated). Asterisks indicate statistical significance between groups (Paired Wilcoxon): **** denotes $p < 0.0001$. (B) Heatmap showing the hierarchical clustering of DMSs associated with genes belonging to pathways in cancer. (C) Principal component analysis of genome-wide DNA methylation profiles in NNK samples. Percentages indicate the variance explained by each principal component.

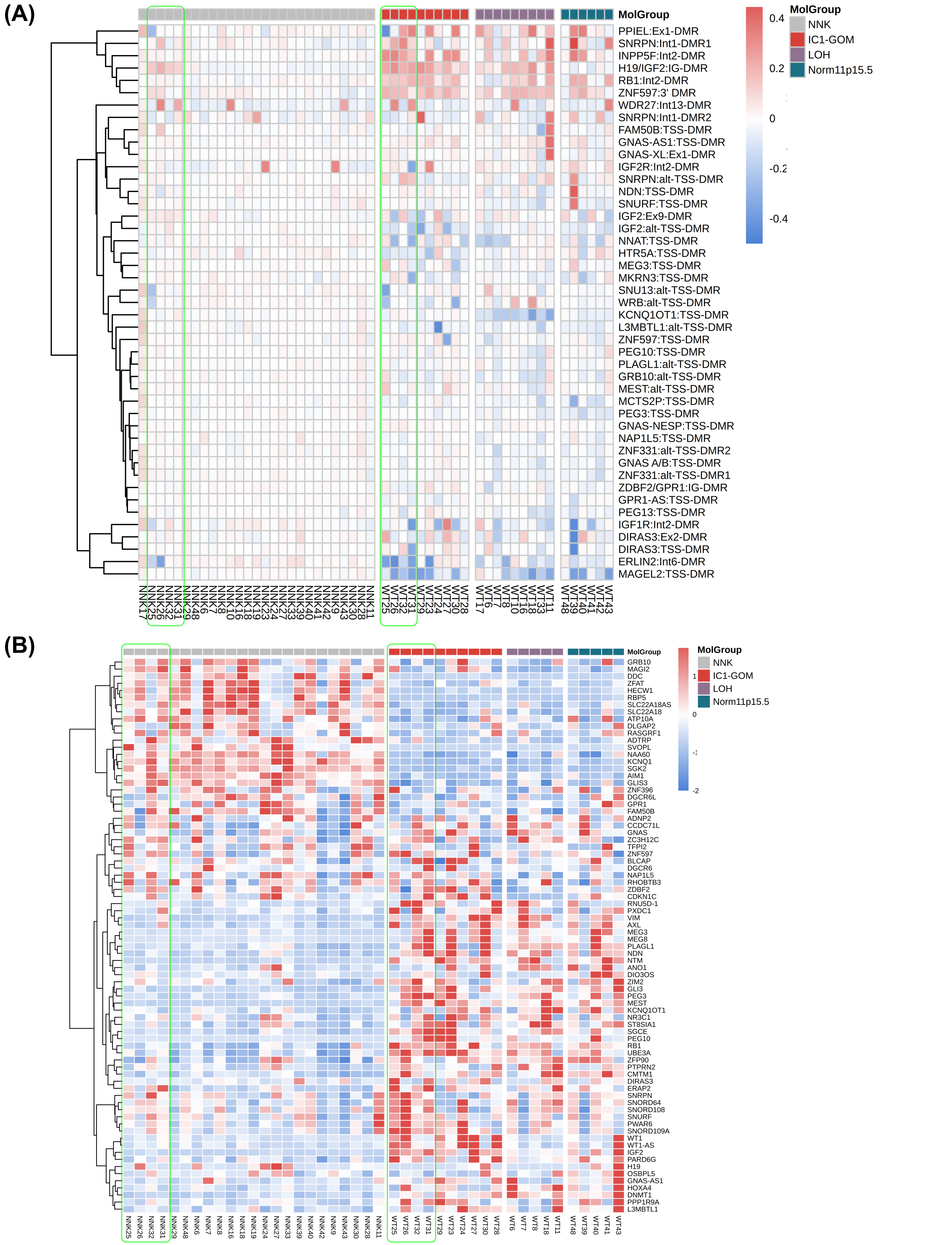

Figure S2. Imprinted methylation and gene expression alterations across 11p15.5 molecular subgroups of WT

Heatmaps showing DNA methylation levels at imprinted differentially methylated regions (DMRs) (A) and expression profiles of imprinted genes (B) across control samples and 11p15.5 subgroups of WTs. Heatmaps represent scaled methylation or expression values, with red indicating higher and blue indicating lower relative levels. Samples from cases with IC1-GOM predisposition are circled in green.

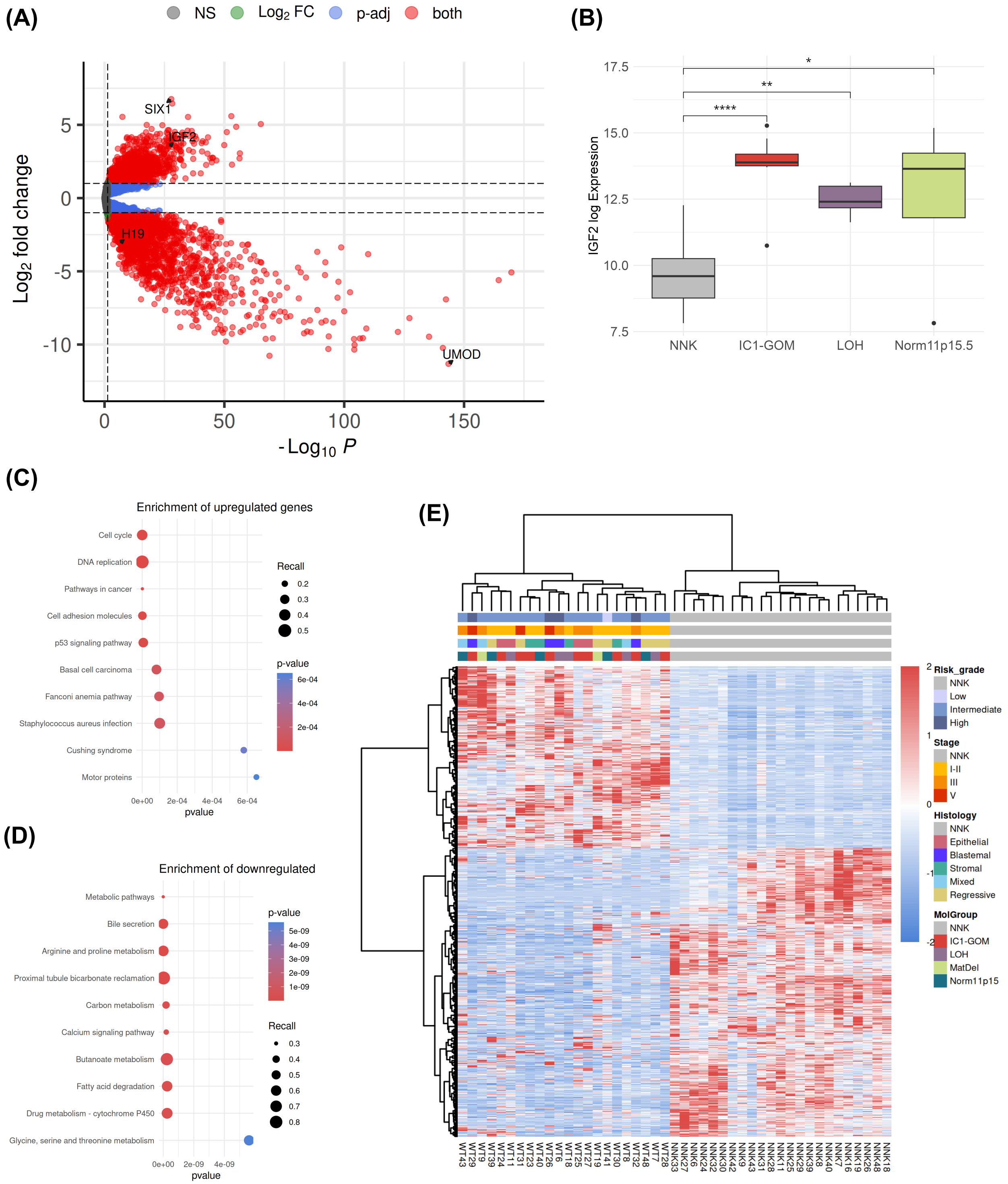

Figure S3. Gene expression and pathway alterations in WT

(A) Volcano plot of differentially expressed genes (DEGs) between WTs and NNKs. Genes meeting both statistical and biological thresholds (adjusted p-value < 0.05 and $|\log_2 FC| \geq 1$) are shown in red. Genes with significant adjusted p-values but lower fold changes are shown in blue, while genes with large fold changes ($|\log_2 FC| \geq 1$) but without statistical significance are shown in green. Genes not meeting either threshold are shown in grey. (B) Boxplots showing $\log_2$-normalized IGF2 expression across 11p15.5 molecular subgroups. Statistical significance is indicated as follows: $*p < 0.05$; $**p < 0.01$; $****p < 0.0001$. Dot plot showing KEGG pathway enrichment of genes upregulated (C) and downregulated (D), respectively. Dot colour reflects adjusted p-value from enrichment analysis (red = higher significance, blue = lower significance), and dot size represents recall, defined as the proportion of genes from a given pathway that are present in the input gene set. (E) Heatmap showing the hierarchical clustering of 4,673 DEGs. Each column represents a sample, and each row a gene.

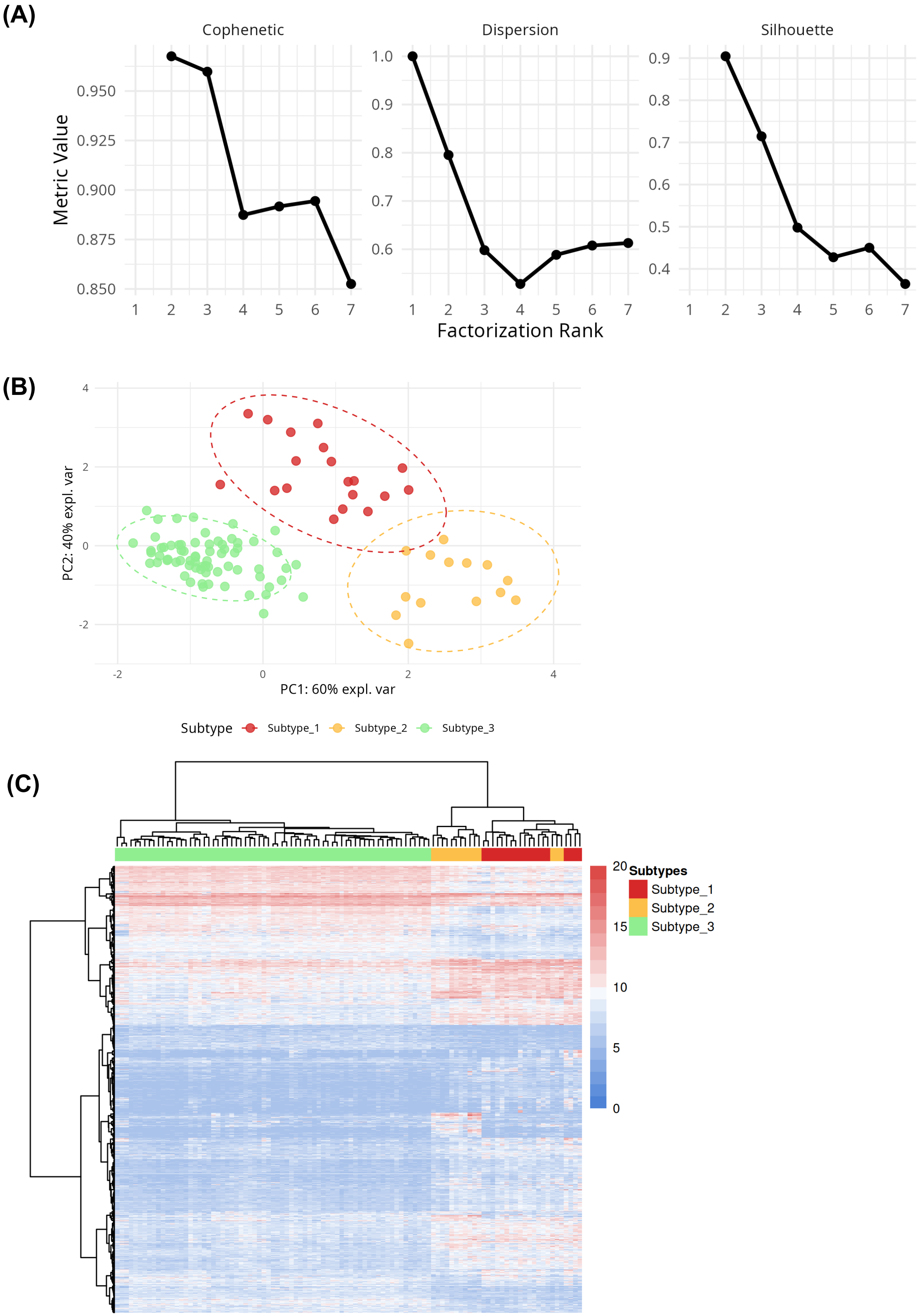

Figure S4. Rank survey analysis and subtype-specific gene template derived from the St. Jude WT cohort's expression data
(A) Multi-facet line plot depicting cophenetic coefficient, dispersion and average silhouette respectively. The metrics (y-axis) were calculated for each tested factorization rank (x-axis) NMF consensus clustering. Higher cophenetic and silhouette values together with lower dispersion indicate more stable and well-separated cluster structures. (B) PCA plot of the coefficient matrix obtained from NMF with rank 3. Each point represents a sample. The separation of points along principal components reflects the distinct cluster assignments derived from the factorization. (C) Heatmap showing the top contributing genes for each of the three subtypes.

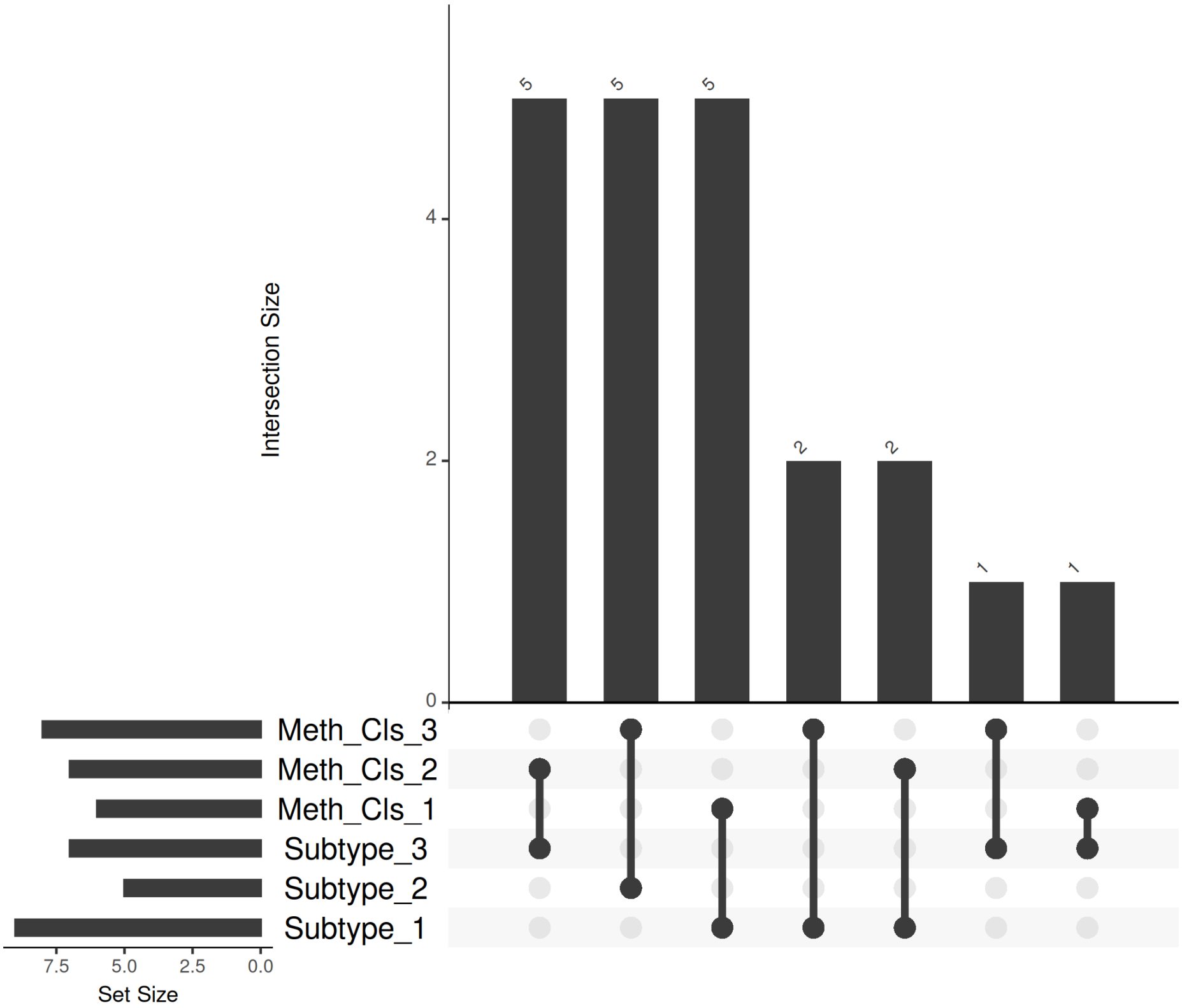

Figure S5. Correlation between gene-expression subtypes and DNA methylation clusters
UpSet plot showing overlap between gene-expression WT subtypes and methylation clusters (21 WT samples, Fisher's exact test adjusted p-value = 0.02296125). The horizontal bar chart (left) shows the sizes of each individual set, while the vertical bar chart (top) displays intersection sizes, indicating the number of samples belonging to specific combinations of subtypes and methylation clusters. Connected dots in the matrix (bottom) represent which sets are included in each intersection.

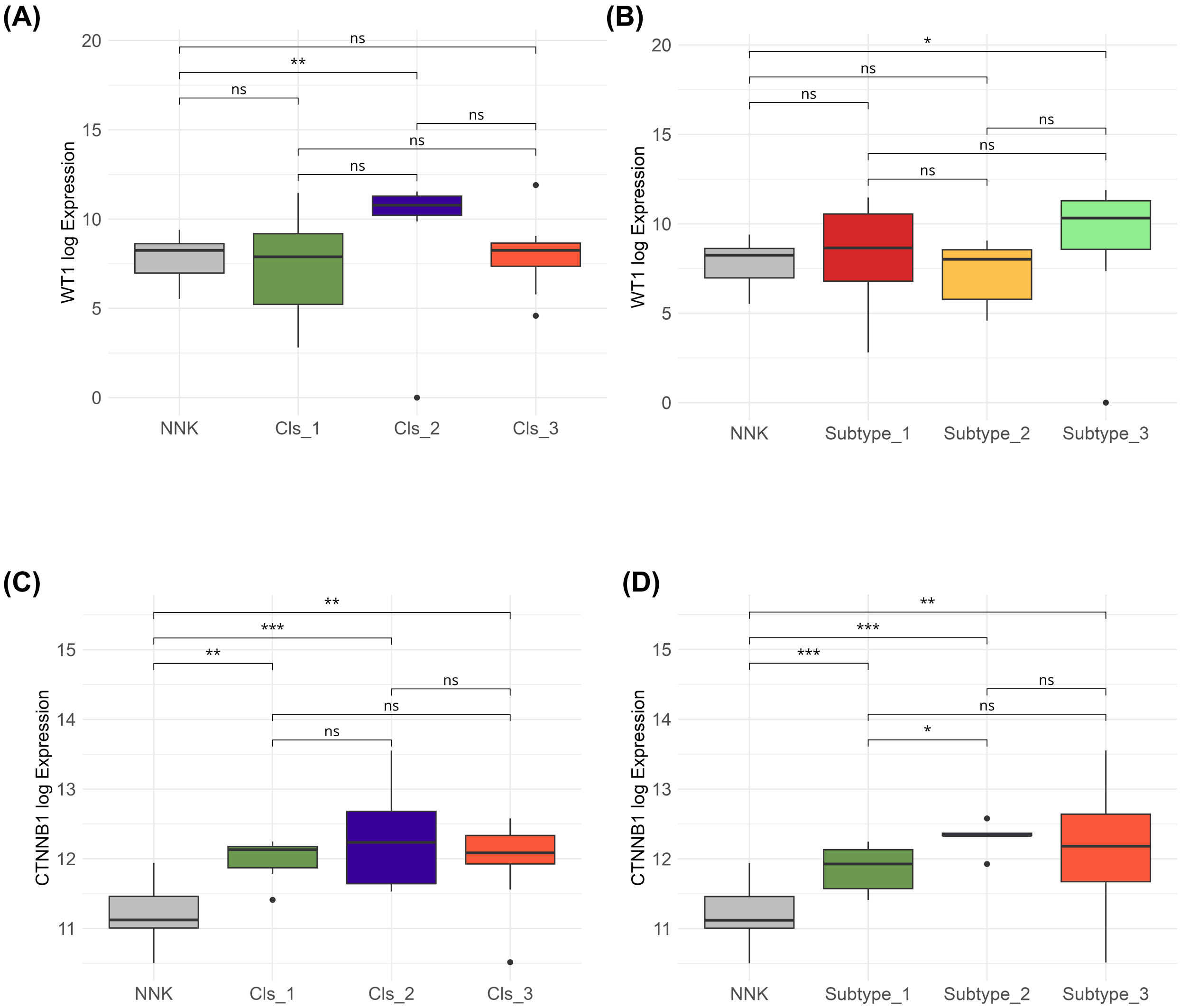

Figure S6. WT1 and CTNNB1 expression across methylation-based clusters and transcriptomic subgroups
(A) WT1 expression across methylation clusters (Cls_1-Cls_3) and (B) transcriptomic subtypes (Subtype_1-Subtype_3). (C) CTNNB1 expression across methylation clusters (Cls_1-Cls_3) and (D) transcriptomic subtypes (Subtype_1-Subtype_3). Statistical significance is indicated as follows: ns, not significant; *$p < 0.05$; **$p < 0.01$; ***$p < 0.001$.

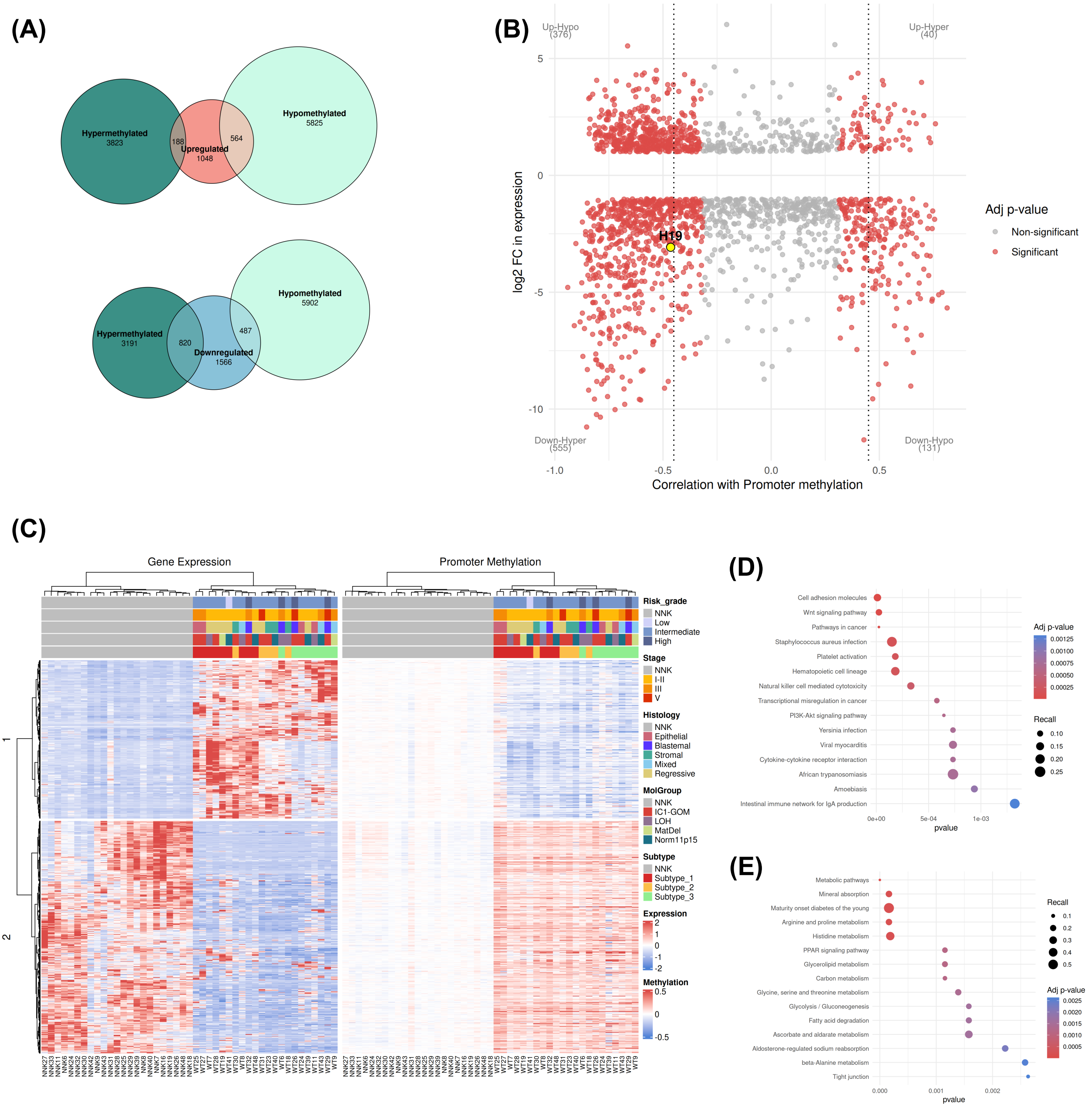

Figure S7. Integrative analysis of DNA methylation and gene expression

(A) Venn diagrams showing the overlap between upregulated DEGs (top) and downregulated DEGs (bottom) with promoter methylation. A gene was considered to be differentially methylated if there was at least three DMSs in its promoter region (5'UTR, TSS1500, TSS200, and 1st Exon). (B) Scatter plot showing the correlation between promoter methylation and gene expression for informative DEGs. The x-axis represents the Spearman correlation coefficient, and the y-axis represents the $\log_2$ fold change in gene expression. Each point represents a gene; dot colour reflects the adjusted p-value of the methylation-expression correlation (red = more significant, blue = less significant). Vertical dashed lines indicate a correlation threshold of ±0.45. (C) Hierarchical clustering of 931 DEGs with significant inverse correlation between gene expression and promoter methylation (left panel) and corresponding promoter methylation levels for the same set of genes (right panel). The clustering order from the expression heatmap is preserved in the methylation heatmap to allow direct visual comparison. Dot plot showing KEGG pathway enrichment of genes significantly upregulated by promoter hypomethylation (D) and significantly downregulated by promoter hypermethylation (E), respectively. The dot colour and size indicate the adjusted p-value and recall, respectively.

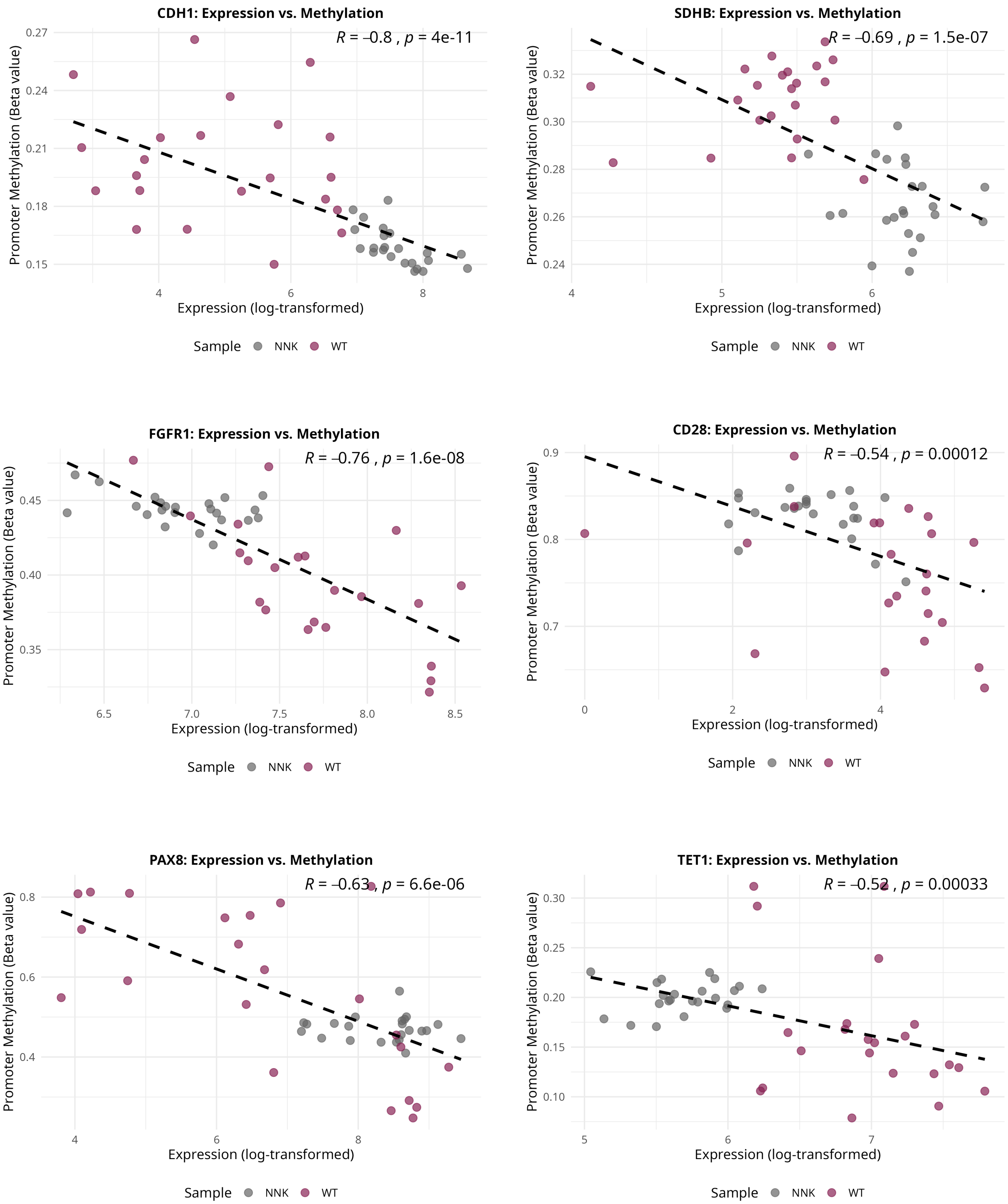

Figure S8. Examples of correlations between cancer gene-expression and promoter DNA methylation
Scatter plots depicting gene-expression and promoter DNA methylation levels for some ncDEGs.

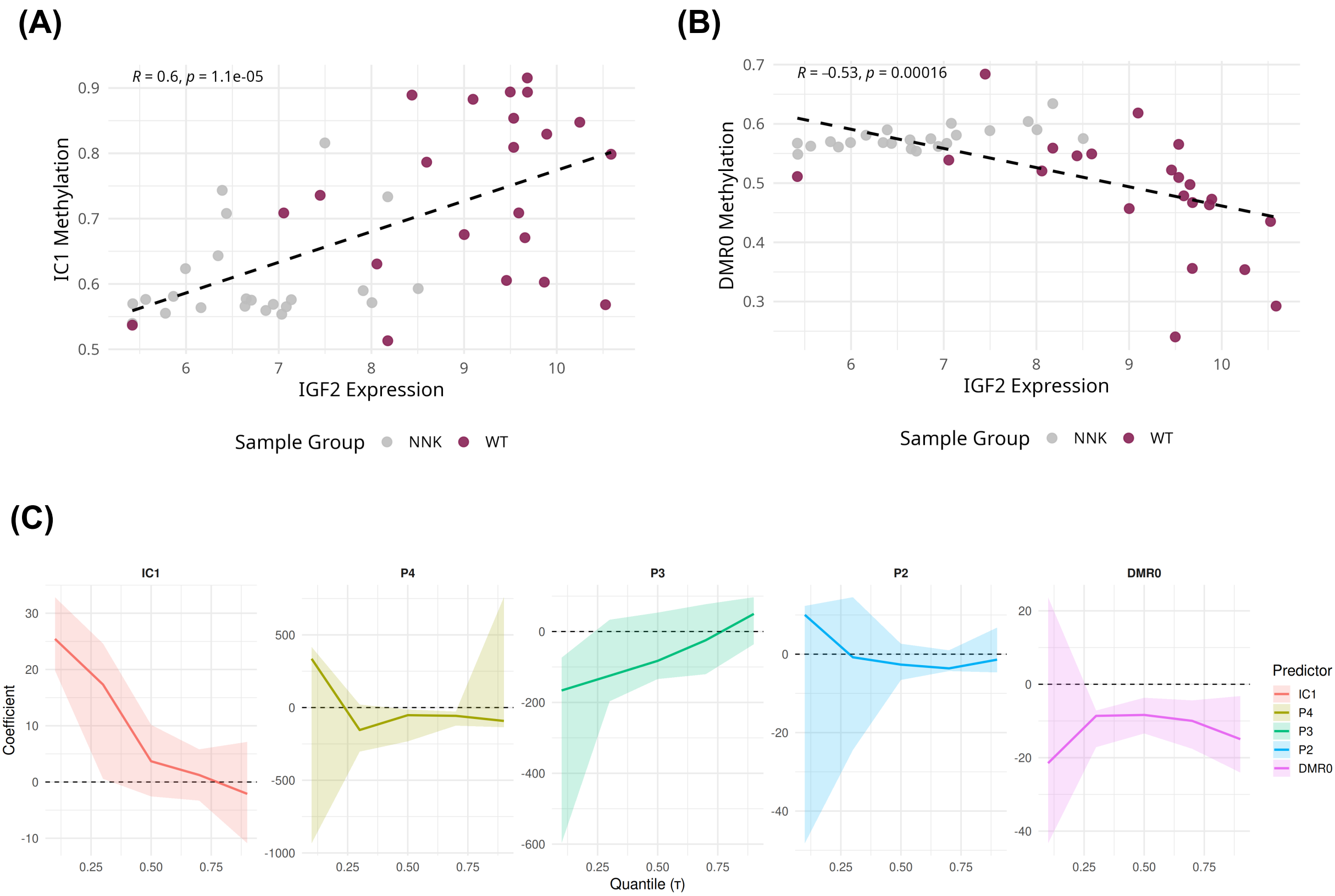

Figure S9. Correlation of IGF2 expression with the DNA methylation of its regulatory regions

Scatter plots depicting the relationship between IGF2 expression (log-transformed; x-axis) and DNA methylation levels (b-value; y-axis) at two key regulatory regions: IC1 (A) and DMR0 (B). Each point represents an individual sample. (C) Multi-facet line plots summarising quantile-regression analysis of IGF2 expression versus methylation at five 11p15.5 loci (IC1, P4, P3, P2 and DMR0). The x-axis shows the expression quantiles ($\tau = 0.1 - 0.9$), and the y-axis gives the regression coefficient for each quantile. Solid lines trace the estimated coefficients and the shaded ribbons denote 95 % confidence intervals. Coefficients above zero indicate a positive association, while coefficients below zero indicate a negative association. Associations are significant where the confidence interval does not include the horizontal zero line.

SUPPLEMENTARY REFERENCES

1. Ritchie ME, Phipson B, Wu D, Hu Y, Law CW, Shi W, Smyth GK. limma powers differential expression analyses for RNA-sequencing and microarray studies. Nucleic Acids Res 2015;43:e47.

2. Gu Z, Hubschmann D. rGREAT: an R/bioconductor package for functional enrichment on genomic regions. Bioinformatics 2023;39.

3. Tarazona S, Garcia-Alcalde F, Dopazo J, Ferrer A, Conesa A. Differential expression in RNA-seq: a matter of depth. Genome Res 2011;21:2213-23.

4. Risso D, Schwartz K, Sherlock G, Dudoit S. GC-content normalization for RNA-Seq data. BMC Bioinformatics 2011;12:480.

5. Love MI, Huber W, Anders S. Moderated estimation of fold change and dispersion for RNA-seq data with DESeq2. Genome Biol 2014;15:550.

6. Kolberg L, Raudvere U, Kuzmin I, Vilo J, Peterson H. gprofiler2 -- an R package for gene list functional enrichment analysis and namespace conversion toolset g:Profiler. F1000Res 2020;9.

7. Gaujoux R, Seoighe C. A flexible R package for nonnegative matrix factorization. BMC Bioinformatics 2010;11:367.

8. Geeleher P, Cox NJ, Huang RS. Clinical drug response can be predicted using baseline gene expression levels and in vitro drug sensitivity in cell lines. Genome Biol 2014;15:R47.

9. Colaprico A, Silva TC, Olsen C, Garofano L, Cava C, Garolini D, Sabedot TS, Malta TM, Pagnotta SM, Castiglioni I, Ceccarelli M, Bontempi G, et al. TCGAbiolinks: an R/Bioconductor package for integrative analysis of TCGA data. Nucleic Acids Res 2016;44:e71.
